# A comprehensive review of Shengdeng in Tibetan medicine: textual research, herbal and botanical distribution, traditional uses, phytochemistry, and pharmacology

**DOI:** 10.3389/fphar.2023.1303902

**Published:** 2023-12-14

**Authors:** Jing Ma, Qiuyue Li, Ting Wang, Hanyu Lu, Jia Liu, Rangji Cai, Yi Zhang, Jing Zhang, Xiaolong Xie, Jinsong Su

**Affiliations:** ^1^ Ethnic Medicine Academic Heritage Innovation Research Center, Meishan Hospital of Chengdu University of Traditional Chinese Medicine, Chengdu University of Traditional Chinese Medicine, Chengdu, China; ^2^ Pharmacy Intravenous Admixture Service of the Affiliated Traditional Chinese Medicine Hospital of Southwest Medical University, Luzhou, China

**Keywords:** Tibetan medicine, Shengdeng, herbal textual research, phytochemistry, pharmacology

## Abstract

“Shengdeng”, a group of Tibetan medicines with diverse biological origins, has long been utilized in Tibet for the treatment of rheumatoid arthritis. It showcases remarkable efficacy in alleviating rheumatism, reducing swelling, and relieving pain. This study aimed to clarify the plant species used as “Shengdeng” and summarize their botanical distribution, traditional uses, phytochemistry, and pharmacology to promote its utilization and development. “Shengdeng” is derived from a remarkable collection of 14 plant species belonging to six distinct families. Extensive phytochemical investigations have led to the identification of 355 chemical constituents within “Shengdeng”. Pharmacological studies conducted on “Shengdeng” have revealed a wide range of beneficial properties, including antioxidant, anticancer, antimicrobial, antiviral, antiparasitic, anti-inflammatory, and anti-arthritic activities. Notably, flavonoids and triterpenoids emerge as the predominant groups among these constituents, contributing to the therapeutic potential and diverse applications of “Shengdeng”. The present review provides a concise summary of the recent advancements in textual research concerning the herbal and botanical distribution, traditional uses, phytochemistry, and pharmacological activities of “Shengdeng”. It is crucial to note that future research on “Shengdeng” should prioritize the analysis of its active ingredients and the establishment of rigorous quality standards. These aspects are essential for ensuring consistency, efficacy, and safety in its clinical application.

## Graphical Abstract

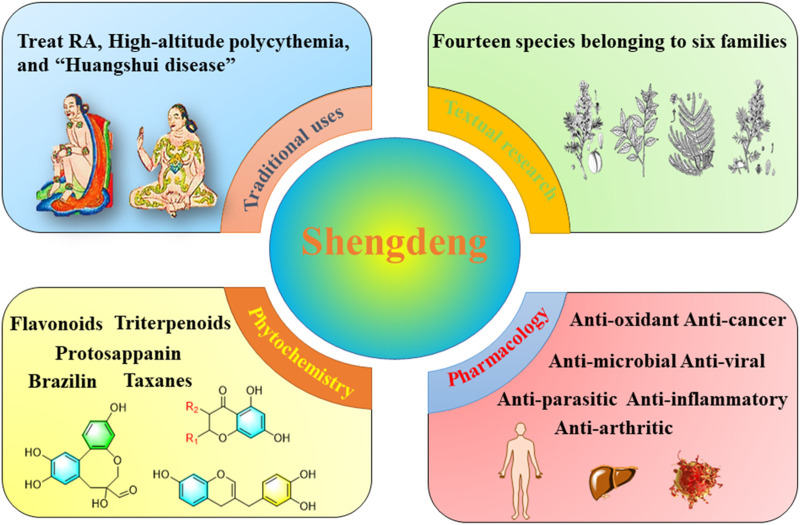

## 1 Introduction

Tibetan medicine stands out for its distinctive theories and treatment methods, which have been refined and perfected through extensive clinical practice by the Tibetan people over centuries. Due to the unique Tibetan medical theory, diseases such as rheumatoid arthritis are considered areas of expertise in Tibetan medicine. In particular, the Tibetan medicine “Shengdeng” has shown significant efficacy in treating rheumatoid arthritis. However, there are numerous alternative names for “Shengdeng”, and its origin is complex, leading to a significant issue of adulteration and improper usage. Therefore, it is necessary to conduct a systematic review and study on the varieties of “Shengdeng” and summarize the current research status.

“Shengdeng”, a collection of herbal medicines with diverse biological origins, holds a significant place in traditional Tibetan medicine. The records of “Shengdeng” can be found in the *Crystal Beads*. Renowned for its efficacy in treating rheumatoid arthritis, “Shengdeng” has attracted considerable attention. The wide distribution of “Shengdeng” in Tibetan regions, coupled with the varying descriptions found in different Tibetan materia medica texts throughout history, has led to confusion regarding its origin. Additionally, the abundance of alternative substitutes further complicates the understanding of Shengdeng’s true source (Zou et al., 2020). In this comprehensive review, we present a meticulous exploration of the herbal and botanical distribution, traditional applications, phytochemistry, and pharmacology of “Shengdeng”. By analyzing the therapeutic potential of this remarkable material in improving human health, our findings contribute valuable insights to guide future research endeavors. This review aims to facilitate a deeper understanding of “Shengdeng” and its multifaceted role in traditional Tibetan medicine, serving as a valuable resource for both researchers and practitioners in the field.

## 2 Textual research of the herbal

**FIGURE 1:**
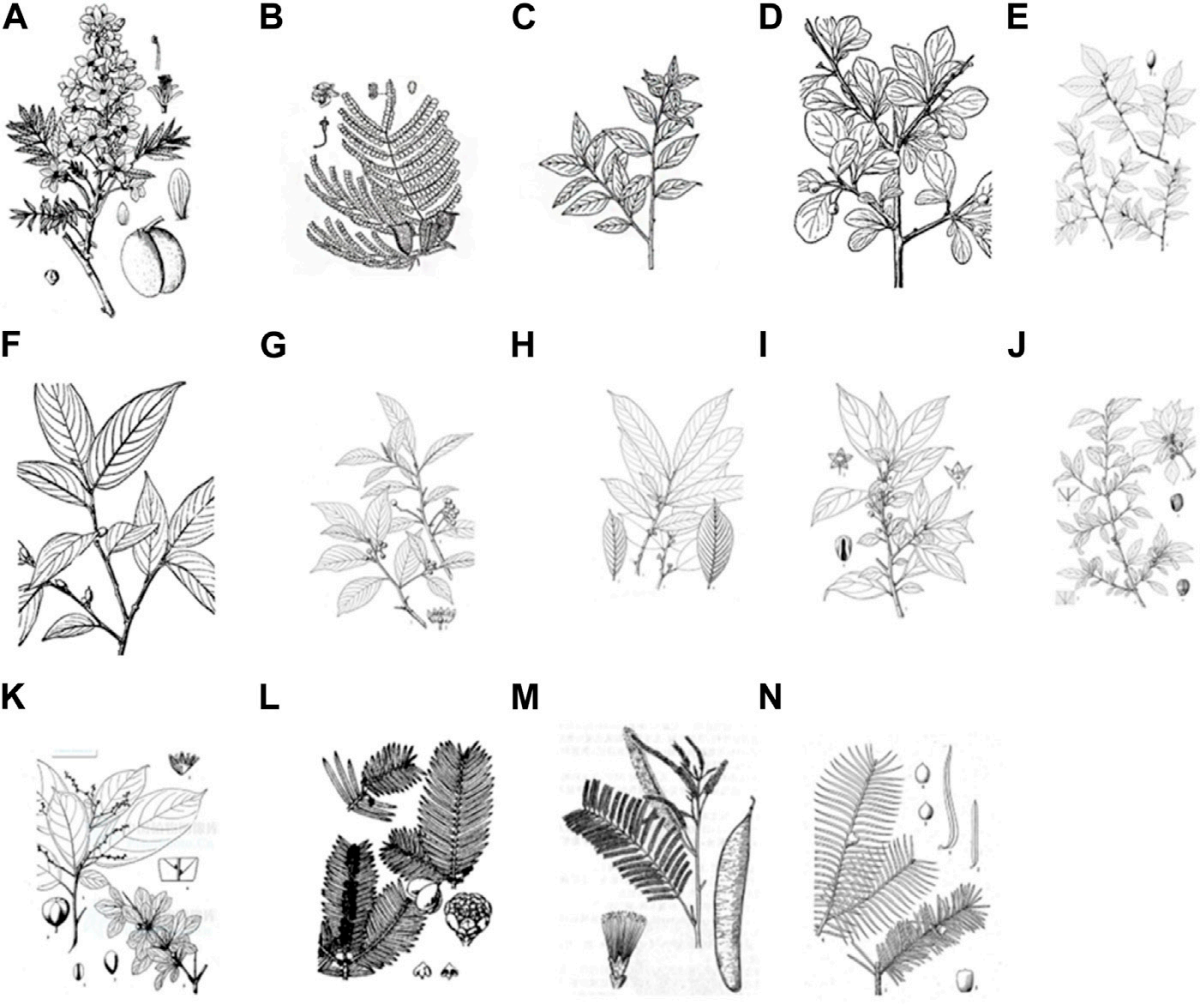
The botanical distribution of “Shengdeng”, **(A)** *Xanthoceras sorbifolium* Bunge. **(B)** *Caesalpinia sappan* L. **(C)** *Rhamnella gilgitica* Mansf. and Melch. **(D)** *Rhamnus parvifolia* Bunge. **(E)** *Rhamnella forrestii* W. W. Sm. **(F)** *Rhamnella martinii* (H. Lév.) C. K. Schneid. **(G)** *Frangula crenata* (Siebold & Zucc.) Miq. **(H)** *Rhamnus sargentiana* C. K. Schneid. **(I)** *Rhamnus xizangensis* Y.L. Chen and P.K. Chou. **(J)** *Rhamnus dumetorum* C. K. Schneid. **(K)** *Rhamnus tangutica* J. J. Vassil. **(L)** *Cephalotaxus sinensis* (Rehder and E.H.Wilson) H. L. Li. **(M)** *Acacia catechu* (L.f.) Willd. **(N)** *Taxus yunnanensis* W. C. Cheng and L. K. Fu. **(C,L,M)** are quoted from Chinese Materia Medica. **(D,F)** are quoted from Atlas of Chinese Higher Plants. **(E,H,I,J,K,N)** are quoted from Flora Reipublicae Popularis Sinicae. G is quoted from Chinese Union of Botanical Gardens.).

Through meticulous textual analysis of historical Tibetan botanical drug records, a fascinating revelation emerges—the authentic Tibetan botanical drug products “Shengdeng” primarily comprise leguminous plant catechins cultivated in subtropical regions encompassing India, Myanmar, Africa, Guangdong, Yunnan, Zhejiang, Guangxi, and Taiwan. Remarkably, the origin, botanical morphology, taste, nature, and therapeutic efficacy of these medicinal materials harmonize impeccably with the corresponding descriptions elucidated in the “Tara Materia Medica”. According to the descriptions, the Crystal beads can be categorized into three distinct groups based on their colors: “Tanhong Shengdeng”, “Bihuang Shengdeng” and “Songbai Shengdeng”. These categories encompass a total of 14 plant species (Li, 2020). In the present review, a series of surveys of herbal texts and research literature was conducted to explore the source of “Shengdeng” and associated plants. The results are summarized in Table 1. The original plant pictures are in Figure 1. (A is quoted from Tibetan Medicine Records. B is quoted from Chinese Tibetan medicine*.* C, L, and M are quoted from *Chinese Materia Medica*. D and F are quoted from *Atlas of Chinese Higher Plants*. E, H, I, J, K, and N are quoted from *Flora Reipublicae Popularis Sinicae*. G is quoted from *Chinese Union of Botanical Gardens*.) (The full botanical plant names have been checked with http://www.theplantlist.org).

**TABLE 1:** The herbal textual research of “Shengdeng”.

NO	Categories	Medicinal material name	Family name	Botanical name	References
1	Tanhong Shengdeng	Wen Guanguo	Sapindaceae	*Xanthoceras sorbifolium* Bunge.	Chinese Tibetan medicine
2	Tanhong Shengdeng	Sumu	Caesalpinia sappan	*Caesalpinia sappan* L.	Chinese Tibetan medicine
3	Bihuang Shengdeng	Xizang Maoru	Rhamnaceae	*Rhamnella gilgitica* Mansf. and Melch.	Crystal Beads
4	Bihuang Shengdeng	Xiaoye Shuli	Rhamnaceae	*Rhamnus parvifolia* Bunge.	Chinese Tibetan medicine
5	Bihuang Shengdeng	Chuandian Maoru	Rhamnaceae	*Rhamnella forrestii* W.W. Sm.	Chinese Tibetan medicine
6	Bihuang Shengdeng	Duomai Maoru	Rhamnaceae	*Rhamnella martinii* (H. Lév.) C.K. Schneid.	New Crystal Beads
7	Bihuang Shengdeng	ChangyeDonglv	Rhamnaceae	*Frangula crenata* (Siebold & Zucc.) Miq.	Chinese Tibetan medicine
8	Bihuang Shengdeng	Duomai Shuli	Rhamnaceae	*Rhamnus sargentiana* C.K. Schneid.	New Crystal Beads
9	Bihuang Shengdeng	Xizang Shuli	Rhamnaceae	*Rhamnus xizangensis* Y.L. Chen and P.K. Chou	New Crystal Beads
10	Bihuang Shengdeng	Cishuli	Rhamnaceae	*Rhamnus dumetorum* C.K. Schneid	New Crystal Beads
11	Bihuang Shengdeng	Ganqing Shuli	Rhamnaceae	*Rhamnus tangutica* J.J. Vassil.	New Crystal Beads
12	Songbai Shengdeng	Cufei	Cephalotaxaceae	*Cephalotaxus sinensis* (Rehder and E.H.Wilson) H.L.Li	Crystal Beads
13	Songbai Shengdeng	Ercha	Fabaceae	*Acacia catechu* (L.f.) Willd	Chinese Tibetan medicine
14	Songbai Shengdeng	Yunnan Hongdoushan	Taxaceae	*Taxus yunnanensis* W.C.Cheng and L.K.Fu	Tibetan medicine crystal mirror herbal medicine

## 3 Botanical distribution

The “Shengdeng” species primarily inhabit the middle and lower altitude regions of the Qinghai-Tibet Plateau in China. The “Tanhong Shengdeng” variety is primarily distributed in high-altitude regions, notably Tibet, Sichuan, Yunnan, and other areas. In contrast, the “Bihuang Shengdeng” type exhibits a wide distribution and is commonly found along the hillside edges of forests. It predominantly flourishes in sub-montane to montane regions. Lastly, the “Songbai Shengdeng” variety thrives at relatively lower altitudes and can be found throughout the entirety of the country (Zou et al., 2020). This group of herbal medicines can be found in up to 12 countries and regions worldwide. In China specifically, the resources of “Shengdeng” are abundant, with presence documented in at least 20 provinces. Notably, the provinces of Sichuan and Yunnan exhibit significant concentrations of these valuable resources (Table 2).

**TABLE 2:** The resource distribution of “Shengdeng”.

Botanical name	Growth altitude	Distribution area/Country
*X. sorbifolium*	2,600∼2,900 m	East Tibet, Southwest Sichuan, and Northwest Yunnan, in China
*C. sappan*	500∼1800 m	India, Myanmar, Vietnam, Malay Peninsula and Sri Lanka, Guangxi, Guangdong, Taiwan, Guizhou, Yunnan and Sichuan in China
*R. gilgitica*	2,600∼2,900 m	Kashmir region in northwestern Himalayas, Southeast Tibet, Northwest Yunnan and West Sichuan in China
*R. parvifolia*	400∼2,300 m	Heilongjiang, Jilin, Liaoning, Inner Mongolia, Hebei, Shanxi, Shandong, Henan, Shaanxi and Mongolia in China, North Korea, Siberia
*R. forrestii*	2000∼3,000 m	Sichuan, northwestern Yunnan and Tibet in China
*F. crenata*	900∼1,200 m	Shaanxi, Henan, Shandong, Anhui, Zhejiang, Jiangxi, Fujian, Guangdong, Guangxi, Hunan, Hubei, Sichuan, and Yunnan in China
*R. xizangensis*	1,600∼3,200 m	Yunnan and Tibet in China
*R.sargentiana*	1700∼3,800 m	Hubei, Chongqing, Sichuan, Yunnan, Tibet and Gansu in China
*R. martini*	800∼2,800 m	Western Hubei, Sichuan, Yunnan, Southeast Tibet, Guizhou and Northern Guangdong in China
*R. dumetorum*	2000∼2,900 m	Sichuan, northwestern Yunnan, Guizhou, Tibet, southeastern Gansu, southern Shaanxi, western Hubei, Jiangxi, Zhejiang and Anhui in China
*R. tangutica*	1,200∼3,700 m	Gansu, Qinghai, Shaanxi, Henan, Sichuan, Tibet in China
*C. sinensis*	600∼2,200 m	Southern Jiangsu, Zhejiang, southern Anhui, Fujian, Jiangxi, Henan, Hunan, Hubei, southern Shaanxi, southern Gansu, Sichuan, southeastern Yunnan, northeastern Guizhou, Guangxi, southwestern Guangdong in China
*A. catechu*	500∼600 m	India, Myanmar, Africa, Guangdong, Yunnan, Taiwan, Zhejiang and Guangxi in China
*T. yunnanensis*	2000∼3,500 m	Yunnan, Sichuan, and Tibet in China, Bhutan, Myanmar

## 4 Traditional uses

In accordance with traditional Tibetan medicine theory, “Shengdeng” holds significant therapeutic value in the treatment of various conditions. It is commonly employed for addressing ailments such as rheumatoid arthritis, high-altitude polycythemia, and “Huangshui disease” in Tibetan medicine (Zou et al., 2020). Some prescriptions containing “Shengdeng” have been clinically tested and modern pharmacological studies have demonstrated their significant anti-inflammatory effects. Several clinical studies have reported the effectiveness of Ershiwuwei ErCha Wan in treating rheumatoid arthritis, highlighting its high application value (Huang et al., 2001; Zha et al., 2017; Liu et al., 2023). These medicinal properties have made “Shengdeng” a prominent ingredient in traditional Tibetan healing practices. We organize the details in Tables 3, 4.

**TABLE 3:** The traditional uses of “Shengdeng”.

No	Local name	Botanical name	Part	Usage, dosage	Traditional uses	Collected, stored and processed	Source
1	Wen Guanguo	*X. sorbifolium*	Xylem of the trunk and branches	Decoction of herbs into soft extracts, Oral: 9–15g, Apply it externally to the affected area or wash the affected area with decoction of botanical drugs	Reduce swelling and pain, dry “Huangshui” in Tibetan medicine, external application of plasters can reduce swelling and cure sores and poison	It is advisable to harvest in spring and summer. Cut off branches or trunks, strip off corks, and divide them into segments or small pieces. Decoction with water, concentrate	Chronicles of Tibetan Medicine, Chinese Tibetan Materia Medica, Chinese Tibetan medicine
2	Sumu	*C. sappan*	Stems, heartwood, flowers, leaves	It is usually used in prescriptions, 8–9 g	Treat fever and vomiting blood, dry “Huangshui” in Tibetan medicine remove blood stasis		Chinese Tibetan medicine
3	Xizang Maoru	*R. gilgitica*	Xylem of the trunk and branches	Decoction of herbs into soft extracts, 9–15 g. Oral: decoction, 4–5g, or into pills, powder. Topical: Make a plaster and apply it to the affected area	Cool the blood, dry “Huangshui” in Tibetan medicine subside a swelling, external application of plasters can reduce swelling and cure sores and poison	It is advisable to harvest in spring and summer. Cut off branches or trunks, strip off corks, and divide them into segments or small pieces. Decoction with water, concentrate	Original interpretation of Crystal Beads, Chronicles of Tibetan Medicine, Chinese Ministry of Health Drug Standards • Tibetan Medicine
4	Xiaoye Shuli	*R. parvifolia*	Xylem of the trunk and branches	Decoction of herbs into soft extracts, 9–15 g	Clear dampness, dry “Huangshui” in Tibetan medicine	It can be harvested all year round, Cut off branches or trunks, strip off corks, and divide them into segments or small pieces. Decoction with water, concentrate	Chinese Ministry of Health Drug Standards • Tibetan Medicine
5	Chuandian Maoru	*R. forrestii*	Xylem of the trunk and branches	Decoction of herbs into soft extracts	Clear dampness, dry “Huangshui” in Tibetan medicine	Decoction with water, concentrate	Original interpretation of Crystal Beads
6	Duomai Maoru	*R. martini*	Xylem of the trunk and branches	Decoction of herbs into soft extracts	Clear dampness, dry “Huangshui” in Tibetan medicine, Wind cold and dampness, leprosy	Decoction with water, concentrate	Original interpretation of Crystal Beads
7	Changye Donglv	*F. crenata*	Stems		Clear dampness, dry “Huangshui” in Tibetan medicine		Chinese Tibetan medicine
8	Duomai Shuli	*R.sargentiana*	Xylem of the trunk and branches	Decoction of herbs into soft extracts	Clear dampness, dry “Huangshui” in Tibetan medicine		Original interpretation of Crystal Beads, Dictionary of Chinese Folk Medicine
9	Xizang Shuli	*R. xizangensis*	Xylem of the trunk and branches	Decoction of herbs into soft extracts	Clear dampness, dry “Huangshui” in Tibetan medicine	Decoction with water, concentrate	Original interpretation of Crystal Bead
10	Ci Shuli	*R. dumetorum*	Xylem of the trunk and branches	Decoction of herbs into soft extracts	Clear dampness, dry “Huangshui” in Tibetan medicine	Decoction with water, concentrate	Original interpretation of Crystal Beads
11	Ganqing Shuli	*R. tangutica*	Xylem of the trunk and branches	Decoction of herbs into soft extracts	Clear dampness, dry “Huangshui” in Tibetan medicine	Decoction with water, concentrate	Original interpretation of Crystal Beads
12	Cufei	*C. sinensis*	Xylem of the trunk and branches, Seeds	Decoction of herbs into soft extracts	The xylem of the branches and twigs cures leprosy, the seeds cure leukemia, lymphosarcoma, five hemorrhoids, digestion, cough, turbidity. External application of plasters can reduce swelling and cure sores and poison	It is advisable to harvest in spring and summer. Cut off branches or trunks, strip off corks, and divide them into segments or small pieces. Decoction with water, concentrate	Chronicles of Tibetan Medicine
13	Ercha	*A. catechu*	Heartwood	Decoction of herbs into soft extracts, dosage: 0.9–3 g	Clear dampness, dry “Huangshui” in Tibetan medicine	Harvested in winter, the botanical drugs are decocted with water, concentrated	Tibetan medicine standards
14	Yunnan Hongdoushan	*T. yunnanensis*	Heartwood, sapwood, branches		Clear dampness, dry “Huangshui” in Tibetan medicine		Tibetan medicine crystal mirror materia medica

**TABLE 4:** Prescription preparation and functional indications of Tibetan medicine “Shengdeng”.

Prescription name	Contains ingredients of “Shengdeng”	Functional indications	Prescription source
Ershiwuwei ErCha Wan	*R. gilgitica*	Treating Arthritis,Anti-inflammatory and Reducing pain, Treating the eczema and scabies	A practical manual for commonly used Tibetan patent medicine in the combination of Tibet and Chinese
	*A. catechu*		Drug Standards of the Ministry of Health of the People’s Republic of China Tibetan Medicine
Ershiwuwei Lvxue Wan	“Shengdeng” concentrate	Treat swelling and pain caused by rheumatism	Tibetan Medicine Standards.
	*R. gilgitica*	Treating the eczema and scabies, Treating Arthritis and Rheumatoid Arthritis	A practical manual for commonly used Tibetan patent medicine in the combination of Tibet and Chinese
Shibawei Ouqu Zhenbao Wan	*R. gilgitica*	Anti-inflammatory and Reducing pain, Treating the eczema and scabies, Treating Arthritis and Rheumatoid Arthritis	A practical manual for commonly used Tibetan patent medicine in the combination of Tibet and Chinese
Ershiyiwei XizangMaoru Wan	*R. gilgitica*	Treating a crooked lower back or swollen joints	Encyclopedia of Chinese Medicine, Tibetan Medicine
Shibawei Dangshen Wan	*R. parvifolia* Bunge	Anti-inflammatory and Reducing pain, Promotes sore healing, Treating the eczema and scabies, Treating Arthritis and Rheumatoid Arthritis	Tibetan medicine standards in Qinghai Province
Liuwei Ximi Wan	*X. sorbifolium*	Treating Arthritis and Rheumatoid Arthritis, Reducing pain, Nourishing kidney, Treatment of renal back pain, frequent urination	New Tibetan medicine formula

## 5 Phytochemistry

In the last few decades, extensive research has led to the isolation and identification of approximately 355 chemical constituents found in the 14 plant species used as “Shengdeng”. These constituents encompass various compound types, including flavonoids, triterpenoids, protosappanin, brazilin, and taxanes. For further details, including the names of the metabolites, their corresponding plant sources, and the references, please refer to Supplementary Table S1.

### 5.1 Flavonoids

**FIGURE 2:**
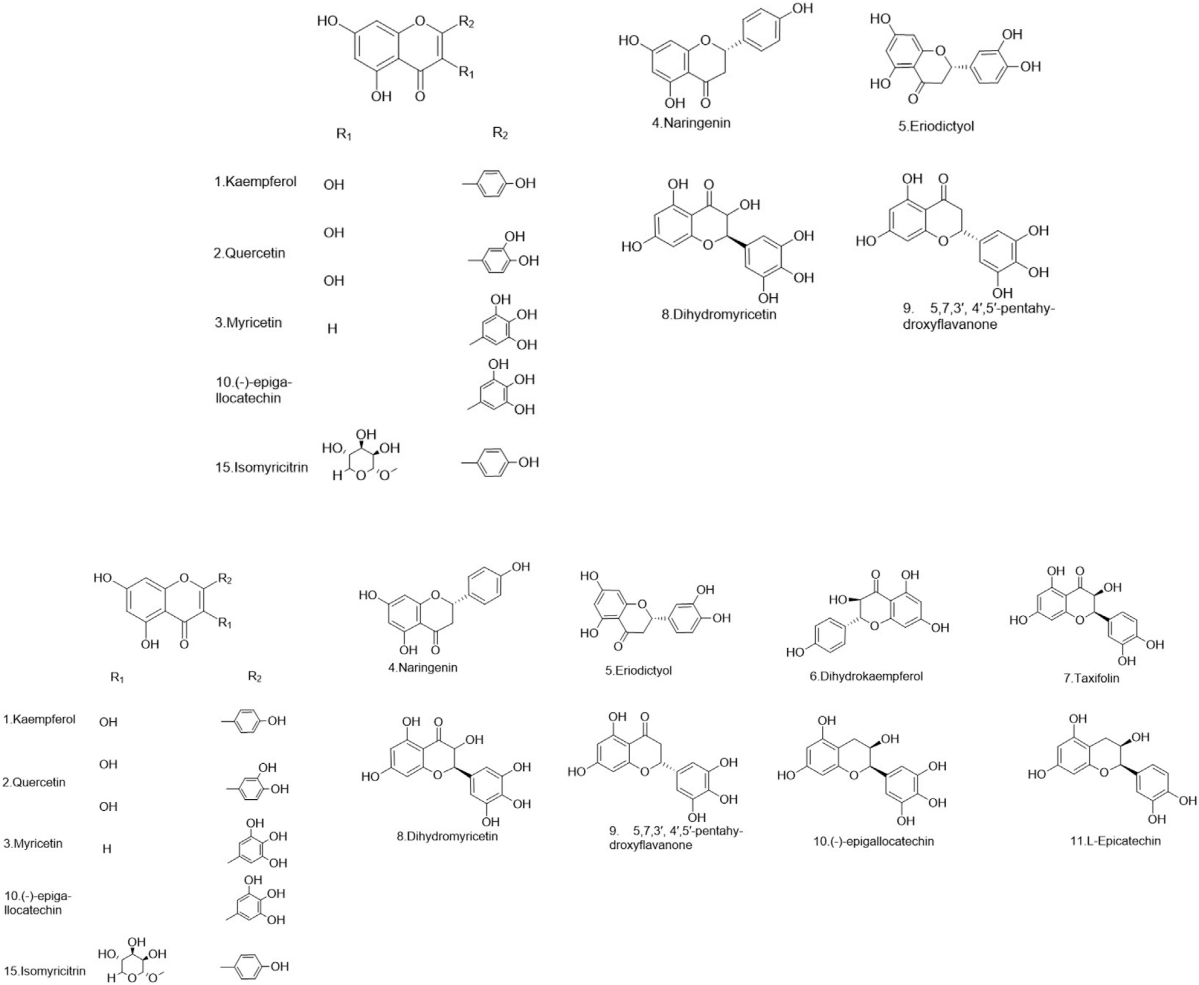
The structures of flavonoids (compound **1–11**).

**FIGURE 3:**
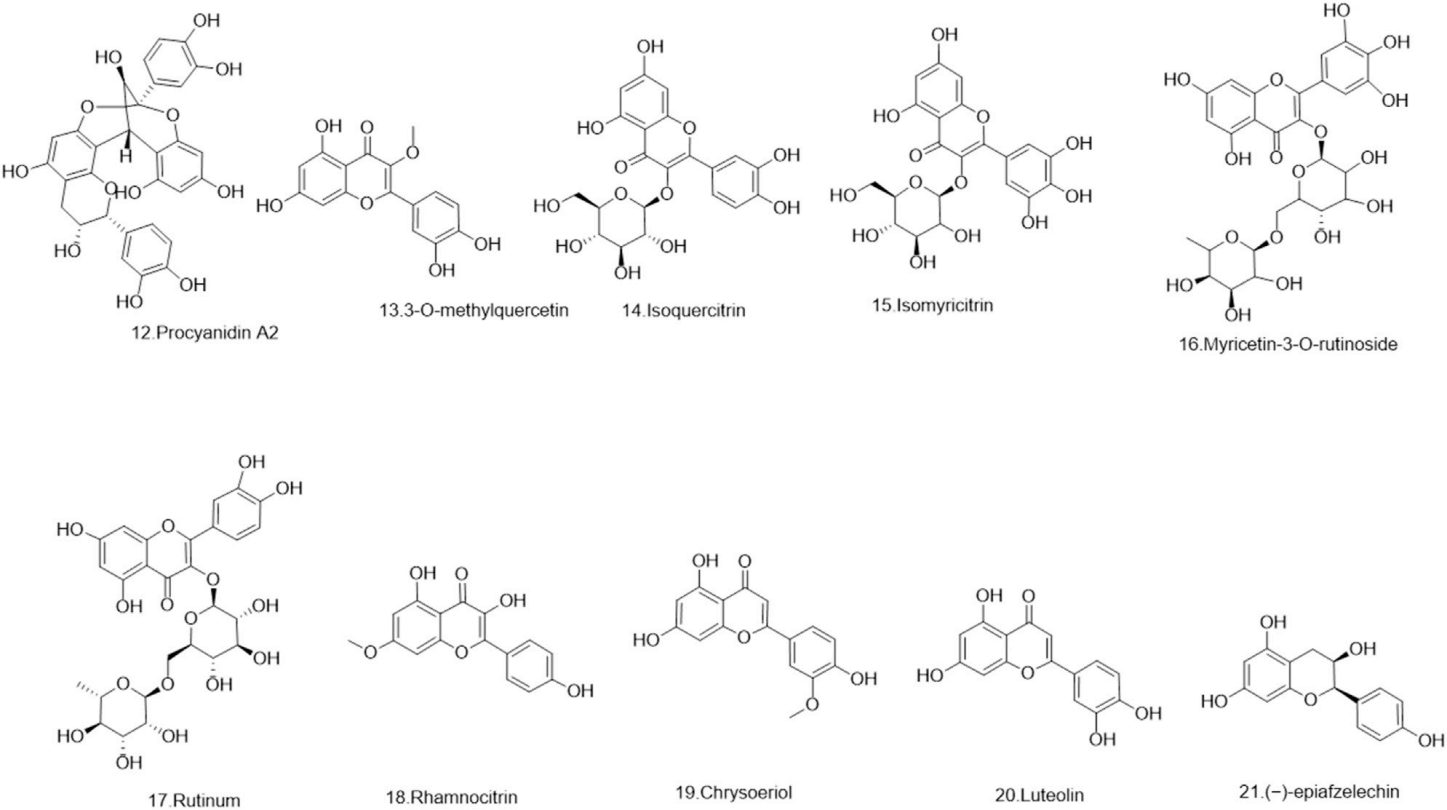
The structures of flavonoids (compound **12–21**).

**FIGURE 4:**
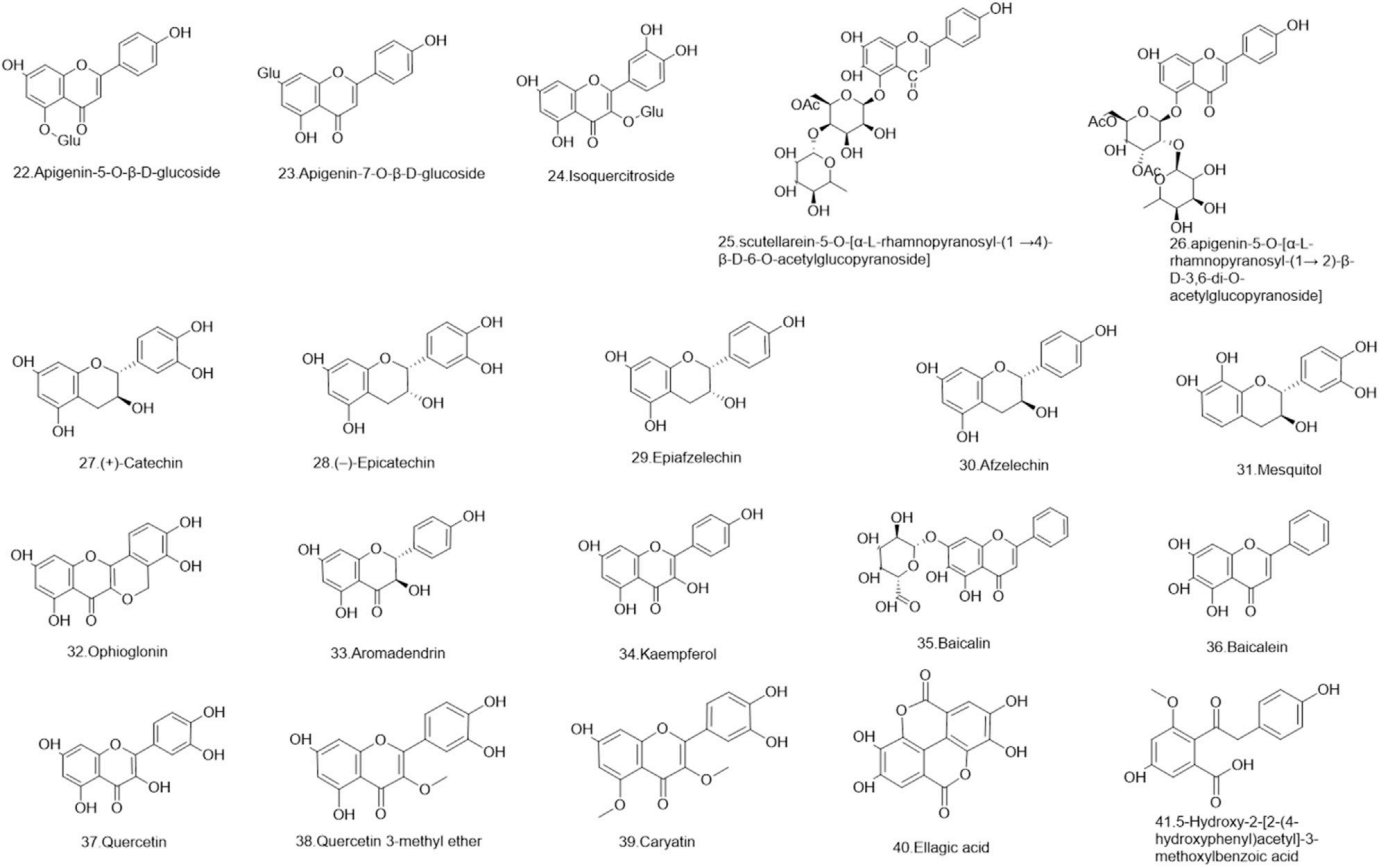
The structures of flavonoids (compound **22–41**).

**FIGURE 5:**
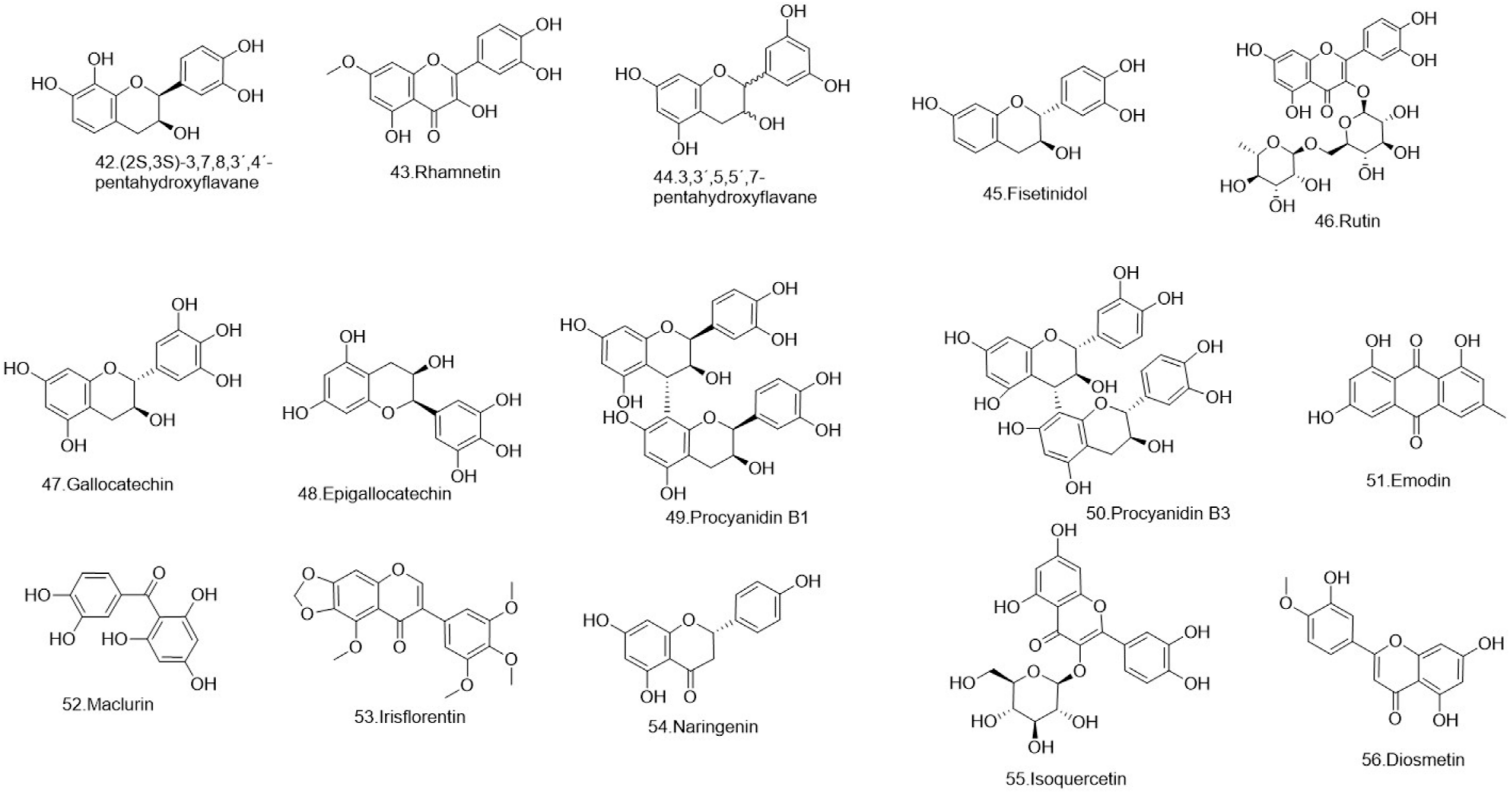
The structures of flavonoids (compound **42–56**).

**FIGURE 6:**
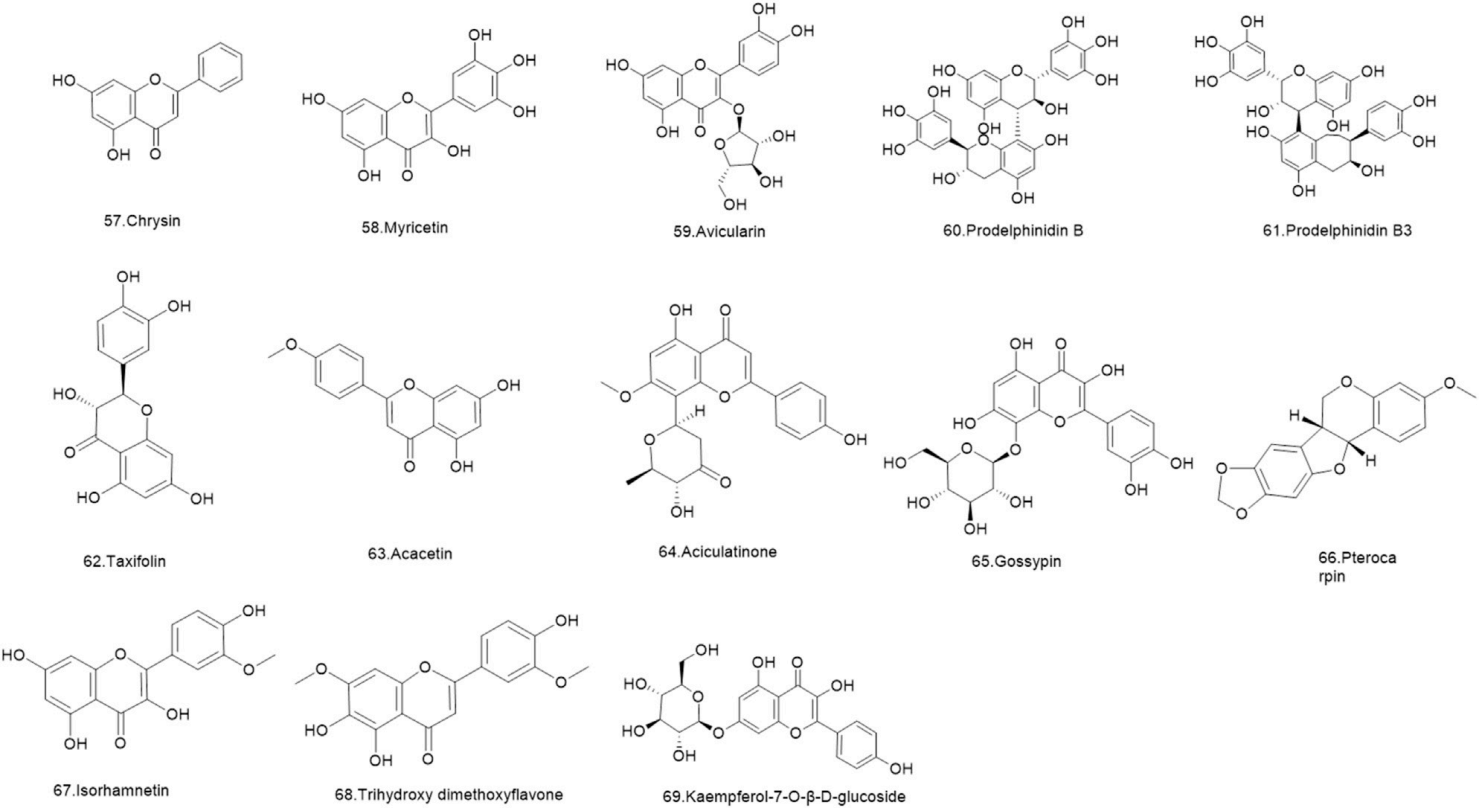
The structures of flavonoids (compound **57–69**).

Flavonoids are a ubiquitous group of naturally occurring polyphenolic metabolites characterized by the flavan nucleus (Peluso et al., 2015). A total of 69 flavonoids **(1–69)** have been reported. 19 flavonoids **(1–19)** have been isolated from *Lignum X. sorbifolium* Bunge (Figures 2–6) (Ni et al., 2009; Yang et al., 2020). Luteolin **(20)** was isolated from the husks of *X. sorbifolium* (Wan et al., 2015). (−)-epiafzelechin **(21)** was isolated from the wood of *X. sorbifolium* (Ma et al., 2004). Flavonoids **(22–26)** were isolated from *Cephalotaxus sinensis* (Rehder and E.H.Wilson) H.L.Li (Li et al., 2007a; Jiang et al., 2013). Flavonoids (**27–68)** were identified from *Acacia catechu* (L.f.) Willd (Li et al., 2010b; Negi et al., 2010; Li et al., 2011; Hong et al., 2015; Adhikari et al., 2021). Kaempferol-7-*O*-β-D-glucoside **(69)**, Kaempferol **(1)**, Quercetin **(2)**, Naringenin **(4)**, Aromadendrin **(33)**, and Taxifolin **(62)** were also isolated from *Rhamnella gilgitica* Mansf. and Melch. (Figure 5) (Pan et al., 1998). Homoisoflavones **(70–120)** were identified from *Caesalpinia sappan* L. (Namikoshi et al., 1987; Wang et al., 2003; Nguyen et al., 2005; Wang, 2006; Shu, 2007; Chen et al., 2008; Wang et al., 2010; Zhao et al., 2010; Cai, 2012; Chen et al., 2012; Tang et al., 2012; Wang, 2013; Zhao et al., 2014; Sheng, 2016; Wang, 2016; Zhou, 2017).

### 5.2 Triterpenoids

**FIGURE 7:**
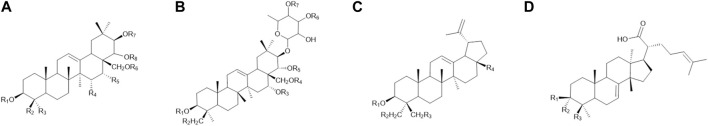
Structural skeletons of triterpenoids from X. sorbifolium.

**FIGURE 8:**
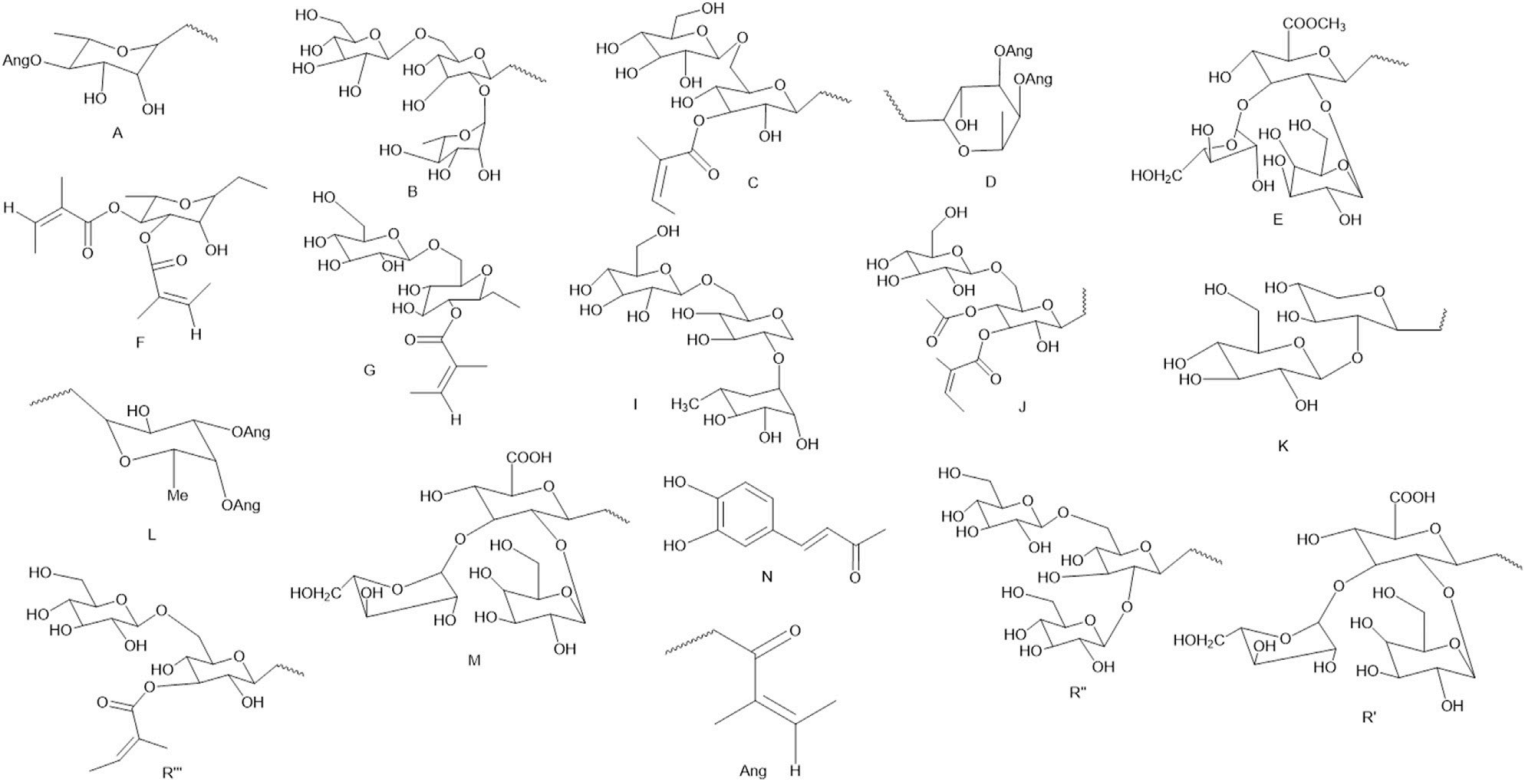
The structure of the code names in the Supplementary Table S1.

**FIGURE 9:**
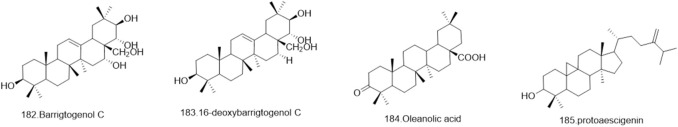
The structures of triterpenoids (compound **182–185**).

Triterpenoids **(121–185)**, also known as ganoderic acid, are widely distributed in nature, which are composed of several isoprene molecules linked end to end without a hydroxyl group. Most triterpenoids contain 30 carbon atoms, and a few with 27 carbon atoms (Gao et al., 2018). *X. sorbifolium* is known to contain triterpenoids as a frequently reported component. Among these triterpenoids, barrigenol-like triterpenoids (A, B) serve as the structural parent cores. Additionally, *X. sorbifolium* also contains lupane triterpenoids (C) and tirucallane triterpenoids (D). Barrigtogenol C, 16-deoxybarrigtogenol C, oleanolic acid, and protoaescigenin were also isolated from *X. sorbifolium* (Figures 7–9; Supplementary Tables S2–S4) (Wan et al., 2013).

### 5.3 Protosappanin

**FIGURE 10:**
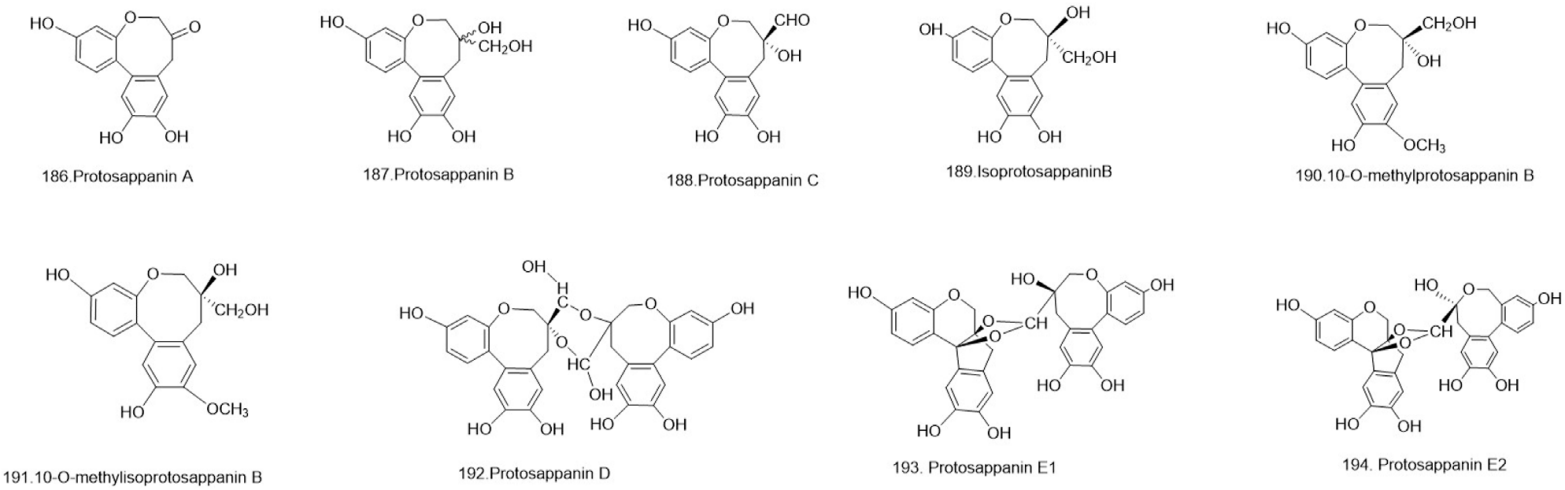
The structures of protosappanins (compound **186–194**).

Nine protosappanin **(186–194)** were isolated from *C. sappan* (Wang et al., 2003; Shu, 2007; Li et al., 2012; Wang, 2016). Among them, 10-omethylprotosappanin B, isoprotosappanin B, and 10-omethylisoprotosappanin B are derivatives derived from protosappanin B. Protosappanin D, on the other hand, is a dimer formed from protosappanin C. Additionally, protosappanin E1 or protosappanin E2 is produced through the polymerization of proto hematoxylin and hematoxylin metabolites. These metabolites represent various structural modifications and polymerizations of protosappanin metabolites, expanding the chemical diversity within this group (Figure 10).

### 5.4 Brazilin

The molecular formula of brazilin is C_16_H_14_O_5_, which is the main active component in *C. sappan*. A total of 13 brazilin metabolites **(195–206)** have been isolated and identified (Xu et al., 1994; Shu et al., 2007; Hung et al., 2009; Cooksey, 2010; Wang and Liang, 2016; Kim and Kim, 2018). Brizilide is not a component naturally present in hematoxylin itself but rather a newly derived component that emerges during the isolation process from brazilin hematoxylin (Supplementary Figure S1).

### 5.5 Taxanes

The taxanes or taxoids are a closely related group of antineoplastic agents that have a unique mechanism of action as inhibitors of mitosis, which are widely used in the therapy of ovarian, breast, lung, esophageal, prostate, bladder, and head and neck cancers. Many taxanes **(208–271)** were isolated from *Taxus yunnanensis* W.C.Cheng and L.K.Fu (Supplementary Figures S2–S5) (Yue et al., 1995; Zhang et al., 1995; Zhong et al., 1996; Zhang et al., 1997; Zhou et al., 1998; Shi et al., 1999; Li et al., 2000; Li et al., 2001; Shinozaki et al., 2001; Li et al., 2002a; Li et al., 2002b; Shinozaki et al., 2002; Li et al., 2003a; Nguyen et al., 2003; Tezuka et al., 2011; Hai et al., 2014).

### 5.6 Other metabolites

In addition to the abovementioned chemical metabolites, several other metabolites have been identified, such as sappanols, flavonoid diglycoside, abietane diterpenoid, and norditerpenoids. Seven sappanols (**272–278)** were isolated from *C. sappan* (Supplementary Figure S6) (Xu et al., 2016; Ahmed et al., 2018; Zhao et al., 2019). A total of 20 metabolites (**279–298)** were isolated from *C. sinensis*, including flavonoid diglycoside, abietane diterpenoid, and norditerpenoids (Supplementary Figures S7, S8). Esters and fatty acid metabolites (**299–313)** were isolated from *A. catechu* (Supplementary Figures S8, S9) (Negi and Dave, 2010; Li et al., 2011; Thakur et al., 2018; Adhikari et al., 2021). Three lignans, seven steroids **(314–323)** and a Polyphenolic **(350)** were isolated from *T. yunnanensis* (Li et al., 2002a; Li et al., 2003b; Hafezi et al., 2020). Twenty cassane diterpenoids **(324–337, 346, 351–355)** and three lignans **(347–349)** were isolated from *C. sappan* (Ma et al., 2015; Tran et al., 2015; Zhu et al., 2017). Alkaloids **(338–345)** were isolated from *C. sinensis* (Supplementary Figure S10) (Ma et al., 2016).

## 6 Pharmacology

**FIGURE 11:**
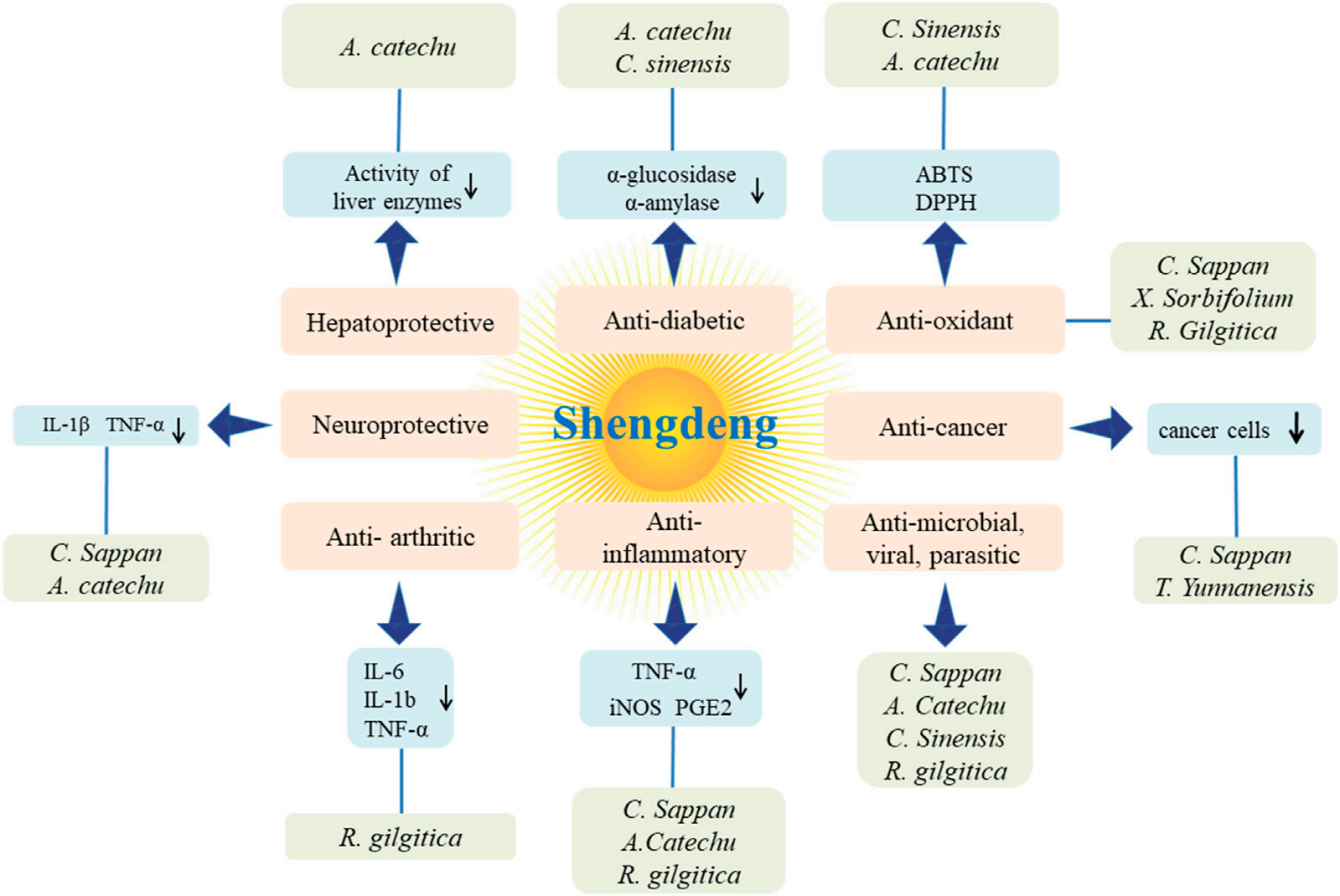
Therapeutic applications of Shengdeng.

The pharmacological studies conducted on “Shengdeng” have consistently shown its remarkable properties, including antioxidant, anticancer, antimicrobial, antiviral, antiparasitic, anti-inflammatory, and anti-arthritic activities, alongside other beneficial characteristics (Figure 11).

### 6.1 Anti-oxidant activity

Among the metabolites isolated from *C. sappan*, including Brazilein (**203**), Sappanchalcone (**272**), Protosappanin A (**186**), Protosappanin B (**187**), and Protosappanin C (**188**), Brazilin (**195**) displayed the highest 2,2-diphenyl-1-trinitrophenylhydrazine (DPPH) free radical scavenging activity (Wetwitayaklung et al., 2005; Sasaki et al., 2007; Batubara et al., 2009). Some findings highlight the antioxidant potential of *X. sorbifolium* and its potential applications in combating oxidative stress (Li et al., 2010a; Zhang et al., 2010). The *in vitro* scavenging activity of the ethanol extract of *R. gilgitica* was assessed, revealing a robust capability to scavenge free radicals and efficiently reduce Fe^3+^ ions (Zhang et al., 2014b; Zhou et al., 2021). The antioxidant activity of various fractions of *C. sinensis* was assessed using DPPH and reducing power assays. The findings demonstrated that *C. sinensis* exhibits significant antioxidant activity (Saeed et al., 2007). Some findings collectively emphasize the exceptional antioxidant properties of *A. catechu*. The regulation of reactive oxygen species and the control of oxidative stress are vital for maintaining cellular balance. Plant extracts, rich in metabolites such as polyphenols, play a significant role in mitigating oxidative stress by demonstrating potent antioxidant activity (Huang et al., 2005; Cai et al., 2006; Hiraganahalli et al., 2012; Saha et al., 2016; Patil and Modak, 2017; Kumar et al., 2018; Babita, 2021a; Adhikari et al., 2021; Babita, 2021b; Shresta et al., 2021). Similarly, the methanol, ethanol, butanol, and water metabolites of *A. catechu* have IC_50_ values ranging from 92.48 to 529.30 μg/mL for the determination of DPPH radicals, ABTS radicals, and superoxide scavengers as well as for the reduction of copper and iron ions, which is primarily due to the presence of Quercetin **(2)**, Kaempferol **(1)**, and Chlorogenic acid **(306)** (Kumar et al., 2019).

### 6.2 Anti-cancer activity

Natural molecules have shown promise in providing potential solutions for combating cancer (Greenwell and Rahman, 2015).

Study demonstrated that *C. sappan* extract can inhibit cancer cell growth by inducing apoptosis and mitochondrial dysfunction in A549 cells (Widodo et al., 2022). The effect of 3-deoxysappanchalcone **(273)** on colon cancer cell growth revealed its inhibitory potential on the activity of T-lymphokine-activated killer cell-originated protein kinase (TOPK). The compound inhibited colon cancer cell proliferation and anchorage-independent cell growth, and it promoted G2/M cell phase arrest and programmed cell death (Zhao et al., 2019b). The apoptotic effect of brazilin **(195)** was confirmed in an *in vitro* model of breast cancer using the MCF-7 cell line. The automated docking tool also demonstrated the therapeutic effect of the brazilin A molecule on the apoptosis inhibitor B-cell lymphoma 2 (BCL-2) protein. This calcium-dependent pathway was mediated through the upregulation of microtubule-associated protein 1A/1B-light chain 2 (LC3-II) and downregulation of P62/SQSTM1 in osteosarcoma cells (Bukke et al., 2018). Using the MG-63 cell line, Kang examined the induction of autophagy by basilicin in osteosarcoma cell cultures and found that this effect was mediated through the Ca^2+^ forkhead box O3A protein (FOXO3A) pathway. In addition, brazilin **(195)** caused autophagic cell death in MG-63 cells by activating phosphorylation at the FOXO3A Ser7 site, initiating nuclear translocation of FOXO3A, and increasing its reporter gene activity, which results in the expression of autophagy-related genes and cell death (Kang et al., 2018). The IC_50_ value of Phanginin R **(346)** from *C. sappan* was detected in the range of 5.3 ± 1.9 to 12.3 ± 3.1 μM, indicating that it has evident cytotoxic effects on lung cancer A549 cells and ovarian cancer cells, as well as A2780 cells. Furthermore, phaginins exhibited the expression of the tumor suppressor protein p53, arrest of the cell cycle in the G1 phase, and initiation of apoptosis in A2780 cells, reflecting their anti-cancer properties (Bao et al., 2016).

Metabolites (**351–354**) exhibited significant inhibition against HL-60 cells (Tran et al., 2015). Studies have shown that brazilin **(195)** isolated from *C. sappan* inhibits BAF phosphorylation *in vitro* and *in vivo*. The results show that brazilin **(195)** is directly related to BAF. The inhibition of BAF phosphorylation leads to abnormal nuclear envelope re-organization and cell death, indicating that the disruption of nuclear envelope re-organization may be a novel anti-cancer therapy. Brazilin **(195)** could be a new cancer drug (Kim et al., 2015b). In another study, various botanical metabolites obtained from ethyl acetate extracts of *C. sappan* showed better anti-cancer activity than the isolated metabolites, indicating that the crude extract was more effective in relieving cancer than molecular extracts (Zhang et al, 2014a).

Recent research has shed light on the potential of metabolites derived from *T. yunnanensis*, such as AgNPs, heteropolysaccharide, and α-conidendrin, in the development of novel anti-cancer therapies (Yan et al., 2013; Xia et al., 2016; Hafezi et al., 2020).

### 6.3 Anti-microbial, anti-viral, and anti-parasitic activity

Some findings suggest that *C. sappan* possesses diverse pharmacological properties, including antibacterial, antiviral, antimalarial, and iron-chelating activities, indicating its potential use in various therapeutic applications (Bukke et al., 2015; Bukke et al., 2015; Puttipan et al., 2018; Safitri et al., 2022).

In another study, from the heartwood of *C. sappan*, numerous neuraminidase inhibitory molecules were isolated, and the maximum inhibitory activity against three types of viral NAs (H1N1, H3N2, and H9N2) was exhibited by a homoisoflavonoid, namely, sappanone A **(70)**, with IC_50_ values of 0.7, 1.1, and 1.0 mM, respectively. The viral neuraminidase (H1N1, H3N2, and H9N2) inhibitory activity of isolated sappanone A **(70)** did not significantly differ from than that of the standard drug oseltamivir with IC_50_ values of 5.8, 5.6, and 1.2 nM, respectively (Jeong et al., 2012). Caesalsappanin G **(332)** and Caesalsappanin H **(333)** isolated from *C. sappan* had a potent antimalarial activity with selectivity indices of 17.6 and 16.4, as well IC_50_ values of 0.78 and 0.52 mM, respectively, which are comparable to the standard compound chloroquine, with IC_50_ value of 0.37 ± 0.02 (Ma et al., 2015). In another study, two novel cassane diterpenes isolated from the seeds of mulberry *C. sappan* extracts were screened for their anti-plasmid activity against the chloroquine-resistant strain K1 of *P. falciparum*. The extracted metabolites Caesalsappanin R **(324)** and Caesalsappanin S **(325)** had a potent antimalarial activity with IC_50_ values of 3.6 and 25.1 mM, respectively, and the standard drug chloroquine has an IC_50_ value of 0.19 ± 0.05. However, the difference was not significant (Zhu et al., 2017).

The aqueous, 50% ethanolic, and butanol extracts of *A. catechu* demonstrated antimicrobial activity against various bacteria, such as *Staphylococcus aureus*, *Pseudomonas aeruginosa, Proteus mirabilis*, *E. coli*, B. and *subtilis* (Patel et al., 2009; Negi and Dave, 2010; Joshi et al., 2011; Dashtdar et al., 2013; Modi et al., 2013; Panya et al., 2019; Shresta et al., 2021). In addition, a study confirmed that drupacine **(338)**, 11-hydroxycephalotaxine **(339)**, cephalancetine A **(340)**, hsocephalotaxine **(341)**, cephalotaxine β-N-oxide **(342)**, 4-hydroxycephalotaxine **(343)**, wilsonine **(344)**, and cephalotaxine **(345)** isolated from *C. sinensis* have excellent activities against tobacco mosaic virus (TMV) and cucumber mosaic virus indoors and outdoors. Its control of TMV is comparable to that of a commercialized anti-viral agent (VA, active ingredient = a mixture of moroxydine hydrochloride and copper acetate). At the same dosage, the inactivation activities against TMV were not significantly different among drupacine **(338)**, 11-hydroxycephalotaxine **(339)**, cephalotaxine **(345)**, and VA (inhibition ratio = 50.76%–53.41%). The inhibited TMV replications of all tested metabolites were inferior to that of VA, but the inhibition ratios of drupacine and cephalotaxine remained greater than 50% (Ma et al., 2016).

### 6.4 Anti-inflammatory activity

In a study, it was demonstrated that the heat-70% EtOH and microwave-70% EtOH extracts of *C. sappan* exhibited significant anti-inflammatory effects. Metabolites derived from *C. sappan*, such as episappanol (**105**), brazilin (**195**), prosapogenin B (**348**), sappanol (**349**), and protosappanin C (**347**), have shown promising potential for treating inflammation (Mueller et al., 2016; Chowdhury et al., 2019). Hematoxylin A, a isoflavonoid derived from *C. sappan*, induces anti-inflammatory effects by inhibiting the production of IL-6, prostaglandin E2 (PGE2), and NO in mouse macrophages. Saponin A inhibits the expression of iNOS and COX-2 in LPS-treated RAW264.7 cells. Furthermore, saponin A exhibits anti-inflammatory effects *in vivo* and protects c57bl/6 mice from LPS-induced death by modulating nuclear factor erythroid 2-related factor 2 (Nrf2) and NF-κB signaling pathways (Lee et al., 2015). In a related study, brazilin could induce an anti-inhibitor of the active groove of NF-κB (IkB) kinase, which targets upstream signaling elements of IkB kinase, thereby promoting formation by disrupting NF-κB activation and signaling complexes at the proximal IL-1 receptor (Jeon et al., 2014). Another study found that NO produced in LPS-induced RAW264.7 cells was scavenged by brazilin and sabanchalcone with IC_50_ values of 10.3 and 31.0 mM, respectively. The results showed that the inhibitory effect of brazilin and saponins on NO production was better than that of indomethacin (IC_50_ value of 46.5). The production of TNF-α and PGE2 was also inhibited by brazilin with IC_50_ values of 87.2 and 12.6 mM, respectively. Hence, the downregulation of the mRNA expression level of TNF-α, iNOS, and COX-2 genes was involved in the anti-inflammatory mechanism of brazilin (Tewtrakul et al., 2015). The anti-inflammatory potential of 3-deoxysappanchalcone **(273)** (3-DSC) in RAW264.7 cells was confirmed. 3-DSC enhances the expression of hemooxygenase-1 (HO-1) at the translational level, thereby activating the phosphatidylinositol 3-kinase mammalian target of the rapamycin (AKT/mTOR) pathway, which contributes to its anti-inflammatory properties. Based on the concept that HO-1 has anti-inflammatory properties, 3-DSC inhibited NO and IL-6 production in LPS-stimulated RAW264.7 cells (Kim et al., 2014).

*A. catechu* extract demonstrates the ability to inhibit the production of important inflammatory cytokines (Burnett et al., 2007; Tseng, et al., 2010; Yimam et al., 2010; Yimam et al., 2012; Sunil et al., 2019). A recent study investigated the anti-inflammatory activities of the 70% ethanol extract of *R. gilgitica* in both RAW264.7 macrophages and rats with complete Freund’s adjuvant (CFA)-induced arthritis (Huang et al., 2016).

### 6.5 Anti-arthritic activity

Rheumatoid arthritis is a prevalent human health problem worldwide. Traditional medicines for RA worldwide have yielded some positive results. Overcoming osteoclastogenesis is considered as an active strategy for the treatment of bone-destroying diseases, RA, and osteoporosis. Brazilin could exhibit anti-inflammatory and chondroprotective effects in chondrocytes and human osteoarthritis cartilage. The antiarthritic effect of brazilin **(195)** was assessed using IL-1b-treated primary chondrocytes, TNF-α, and IL-1b-treated cartilage explants. The loss of glycosaminoglycan from cartilage explants stimulated with IL-1b and TNF-α reduced after Brazilin **(195)** treatment, and the anti-inflammatory activity was evident through the regulation of NFKB1/p50. In chondrocytes, basilicin inhibited the IL-1b-induced inhibition of osteoarthritis markers by inducing NFKB1/p50, indicating a chondroprotective effect (Weinmann et al., 2018). The acute inflammatory paw edema and arthritis index were found to be reduced by brazilin **(195)** in an *in vivo* model of arthritis using CIA mice (Jung et al., 2015a). Microstructural studies have shown that brazilin **(195)** treatment significantly increases bone density, prevents joint destruction and surface wear, and improves bone formation. In addition, serum concentrations of inflammatory cytokines such as IL-6, IL-1b, and TNF-α were attenuated by brazilin **(195)** treatment. In another study, the anti-inflammatory, bone-protective, and anti-RA activities were proven by evaluating the effect of Sappanchalcone **(272)** in CIA-presented male DBA/1J mice. Based on the aforementioned studies, the anti-arthritic activity of brazilin **(195)** (10 mg/kg) and Sappanchalcone **(272)** (10 mg/kg) was similar to that of standard drug methotrexate (3 mg/kg) (Jung et al., 2015a; Jung et al., 2015b). The opposite effect of brazilin **(195)** on osteoclast differentiation confirmed that brazilin **(195)** dose-dependently inhibited the receptor activator of nuclear factor Kpa-B ligand (RANKL) to promote osteoclast differentiation of RAW264.7 cells without signs of cytotoxicity. Brazilin **(195)** reduced RANKL-induced NF-κB p65 phosphorylation, extracellular signal-regulated kinases, and the appearance of inflammatory negotiator genes (TNF-α, INOS, IL-6, and COX-2) in RAW264.7 cells, indicating its therapeutic effect in avoiding bone loss (Kim et al., 2015a). Another study has proposed that the anti-rheumatoid arthritis effect of the ethyl acetate extract of *R. gilgitica* (RGEA) may be linked to its capacity to promote apoptosis and inhibit the inflammatory response, potentially by modulating the JAK-STAT pathway (Su et al., 2021). A study showed that the Tibetan medicine Qi-Sai-Er-Sang-Dang-Song Decoction inhibits TNF-α-induced rheumatoid arthritis in human fibroblast-like synoviocytes via regulating NOTCH1/NF-κB/NLRP3 pathway (Su et al., 2023).

### 6.6 Neuroprotective activity

Neurodegenerative diseases, including Alzheimer’s disease and Parkinson’s disease, present a significant health challenge in industrialized countries. These diseases are characterized by microglial activation and subsequent neuroinflammatory responses (Rajput et al., 2020; Singh and Devasahayam, 2020). The ethanolic extract of *C. sappan* has demonstrated neuroprotective and anti-cerebral ischemic effects in an experimental model (Wan et al., 2019). Protosappanin A **(186)** reversed the neuroinflammatory effect of BV2 cells under the action of LPS by significantly inhibiting the production of IL-1β and TNF-α. In addition, Protosappanin A **(186)** dose-dependently decreased the messenger ribonucleic acid (mRNA) expression of monocyte chemoattractant protein 1 (MCP-1), IL-1β, and IL-6. Moreover, Protosappanin A **(186)** inhibited the inflammatory pathway of LPS treatment by downregulating JAK2 and STAT3 phosphorylation and STAT3 nuclear translocation (Wang et al., 2017a). *In vitro* studies using the thioflavin-T fluorescence assay and transmission electron microscopy demonstrated that hematoxylin significantly reduced the cytotoxicity induced by Aβ42 by inhibiting the formation of Aβ42 fibrils (Tu et al., 2016). Brazilein (**203**) has been shown to reverse the elevated expression of TNF-α and nucleotide-binding oligomerization domain-containing protein 2 (NOD2) induced by cerebral ischemia and reperfusion in mice (Xiao et al., 2016).

### 6.7 Hepatoprotective activities

Study showed that ethyl acetate extract of *A. catechu* (250 mg/kg) can inhibit the toxicity of liver injury in tetrachloride-induced albino rat using biochemical (measurement of serum transaminases, serum alkaline phosphatase, and serum bilirubin) and histopathological assessment (Jayasekhar et al., 1997). *A. catechu* herbal extracts were demonstrated as hepatoprotective with IC_50_ of 114.8 μg/mL on HepG2 cells toxified with *tert*-butyl hydroperoxide (*t*-BH). The anti-oxidant potential of this plant is attributed to its hepatoprotective activity by reducing lipid peroxidation and cell damage (Hiraganahalli et al., 2012). Similarly, plant ethyl acetate extract showed significant hepatoprotective ability in an *in vivo* model (Ray et al., 2006). Moreover, in a Wistar rat model experiment, *A. catechu* seed and bark extracts exhibited hepatoprotective effects, which were related to the decrease of the activity of liver enzymes (alanine aminotransferase, alkaline phosphatase, and aspartate aminotransferase) by reducing lipid peroxidation, and enhanced anti-oxidant activity by increasing glutathione and increasing the activity of peptides and superoxide dismutase (Lakshmi et al., 2018).

### 6.8 Anti-diabetic activities

The extracts of *A. catechu* has shown an anti-diabetic activity with IC_50_ of 49.9 μg/mL toward porcine pancreatic α-amylase and 0.4977 mg/mL against α-glucosidase (Khadayat et al., 2020). Another study found that the methanol extract of *A. catechu* inhibited α-amylase with IC_50_ of 49.9 ± 0.4 μg/mL, and kinetic studies indicated that the extract exhibited mixed-type inhibition (Khadayat et al., 2020). A study showed that feeding with an ethanolic extract of catechins on streptozotocin (STZ-)-induced diabetic rats increased their glucose tolerance by 22% and 27% after 7 and 14 days, respectively, whereas those fed a low-dose STZ showed significantly increased glucose tolerance (Swayam, 2011). Catechin **(27)**, Epicatechin **(28)**, Gallocatechin **(47)**, Epigallocatechin **(48)**, and Procyanidin B1 **(49)**were identified from *A. catechu*, which exerted anti-diabetic effects by reducing the activities of α-glucosidase, α-amylase, and aldose reductase (Adhikari et al., 2021).

The hypoglycemic effect of *C. sinensis* leaves was studied in STZ-induced diabetic rats. The results show that *C. sinensis* leaf extract is a potential drug for treating diabetes, and its active ingredients include flavonoids (Li et al., 2007c) Furthermore, previous investigation suggested that *C. sinensis* extract has good hypoglycemic and hypolipidemic effects, which may be beneficial to hyperglycemia and may decrease HDL (Muhammad et al., 2013).

### 6.9 Toxicity

The historical use of “Shengdeng” as a traditional medicine for thousands of years indicates its long-standing reputation as a safe therapeutic option. Ancient texts documenting the use of “Shengdeng” do not mention any instances of toxicity associated with its usage.

### 6.10 Other effects

A study has found the phenols from the leaves of *X. sorbifolia* could be used as natural neuroinflammation inhibitors (Li et al., 2016). Another study has shown that barrigenol-like triterpenoids derived from *X. sorbifolia* husks exhibited s significant inhibitory activity against the proliferation of three human tumor cell lines, namely, HepG2, HCT-116, and U87-MG (Wang et al., 2017b). Furthermore, a study indicated that the husk of *X. sorbifolia* might prevent inflammation-related neurodegenerative disorders by controlling the expression of the nuclear NF-κB signaling pathway, which clearly inhibited LPS-induced NO production in BV-2 cells (Zhao et al., 2022).

*C. sinensis* extracts have the potential to be developed into herbal products with hepatoprotective and nephroprotective properties (Saeed et al., 2008). Some fundings suggest that the aqueous extract of *A. catechu* has immunomodulatory effects on both cell-mediated and humoral immunity (Ismail and Asad, 2009).

## 7 Conclusion and perspectives

According to Tibetan medical documents, a total of 14 species were used as “Shengdeng”. Extensive phytochemical investigations have resulted in the identification of 355 chemical constituents within “Shengdeng”. The pharmacological studies conducted on “Shengdeng” have unveiled a diverse array of beneficial properties, including potent antioxidant, anticancer, antimicrobial, antiviral, antiparasitic, anti-inflammatory, and anti-arthritic activities. However, it is important to acknowledge that there are still several unresolved issues that require further investigation and clarification in future research endeavors. These areas of focus will contribute to a more comprehensive understanding of the therapeutic potential and mechanisms of action of “Shengdeng”.

Firstly, as a representative multi-origin Tibetan medicine, “Shengdeng” exhibits inherent variations in chemical composition among different plant sources, leading to heterogeneity in the content of active constituents. This inherent variability may significantly impact the therapeutic efficacy and safety profiles of the medication (Kelsang et al., 2023). Secondly, although “Shengdeng” derived from different plant sources may share similar pharmacological effects, the inherent variations in botanical origin and growth conditions can give rise to nuanced pharmacodynamic profiles or even distinct therapeutic outcomes. Consequently, the evaluation of Shengdeng’s therapeutic efficacy and the design of its optimal formulations are confronted with intricate challenges. Thirdly, the research on multi-origin “Shengdeng” confronts the critical issue of establishing harmonized and comprehensive standards. The diverse array of plant species and their inherent differences necessitate the development of standardized methodologies and evaluation frameworks that can accommodate the unique characteristics of multi-origin plants. Achieving such standardization is pivotal to ensure consistent quality control and efficacy assessment. Lastly, Ancient texts documenting the use of “Shengdeng” do not mention any instances of toxicity associated with its usage. Modern pharmacological studies have not reported any side effects or toxicity at present. Therefore, further research on “Shengdeng” in this direction would be worthwhile.

Given the aforementioned challenges in the research of multi-origin Tibetan medicine “Shengdeng”, future investigations should focus on the following key areas. Firstly, it is crucial to conduct comprehensive chemical analysis and comparative studies on “Shengdeng” derived from different plant sources to identify the major bioactive metabolites and assess their quantitative variations. Innovative approaches such as multidimensional separation techniques like comprehensive two-dimensional liquid chromatography (2D-LC) should be employed to achieve improved separation and qualitative-quantitative analysis of the complex constituents in multi-origin “Shengdeng” (Bo et al., 2023). Secondly, establishing scientific quality control methods for “Shengdeng” is essential to ensure the herbal material’s quality. This involves developing standards and protocols for collection, storage, processing, and strengthening regulatory oversight and management of the entire herbal production process to ensure the stability of Shengdeng’s quality (Peng et al., 2022). Thirdly, conducting clinical research and experimental pharmacological evaluations are necessary to gain a deeper understanding of the variations and consistency in the pharmacological effects of multi-origin plants. Based on the research findings, suitable dosage forms and treatment regimens can be designed for different plant sources to enhance the consistency and controllability of therapeutic efficacy (Wang et al., 2022). Fourthly, it is imperative to establish standardized methods and evaluation systems specific to multi-origin plants like “Shengdeng” to ensure comparability and reproducibility in research and application. Developing a unified set of standards and guidelines encompassing plant collection, quality assessment, component analysis, and pharmacological evaluation is essential (Zhong et al., 2022). Lastly, Toxicity and side effects were evaluated using modern pharmacological methods in relevant animal models. These include safety pharmacology, genetic toxicology, acute and subchronic toxicology, absorption, distribution, metabolism and excretion (ADME) studies, reproductive and developmental toxicity, and carcinogenic potential assessments (Cai and Suo, 2023).

This review presents a comprehensive overview of the latest advancements in the textual research of “Shengdeng”, encompassing its herbal and botanical distribution, traditional uses, phytochemistry, and pharmacological activities. As an integral component of Tibetan medicine, “Shengdeng” holds significant medicinal value and is widely employed in clinical settings. By providing a comprehensive understanding of the plant species utilized as “Shengdeng” and their applications, this review contributes to the existing knowledge in the field and serves as a valuable resource for researchers and practitioners alike.
