# Supplementary figures and images for: A comprehensive review of Shengdeng in Tibetan medicine: textual research, herbal and botanical distribution, traditional uses, phytochemistry, and pharmacology
Source: Front Pharmacol. 2023 Dec 14;14:1303902. doi: 10.3389/fphar.2023.1303902 (PMC10762315; doi:10.3389/fphar.2023.1303902)

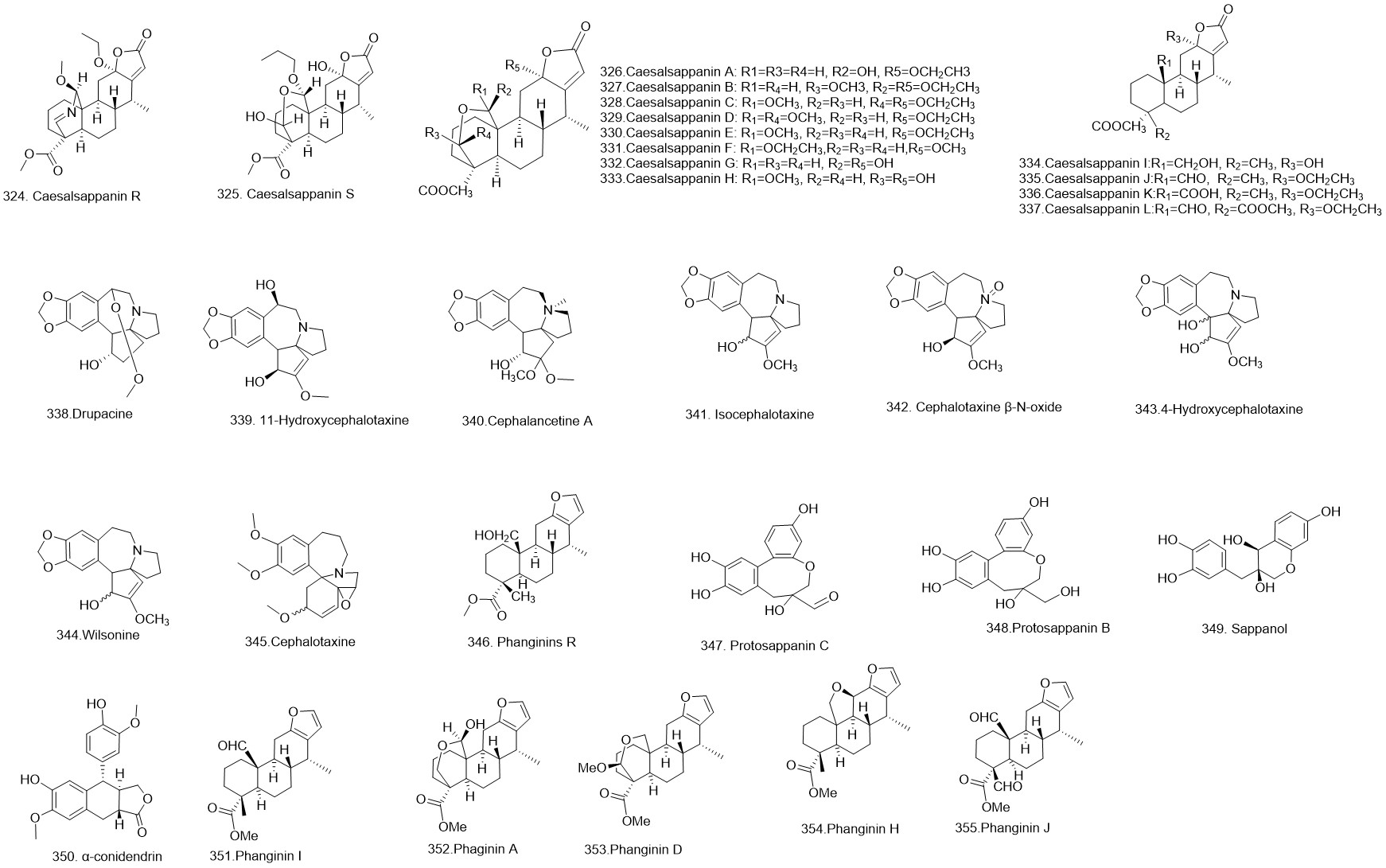

Supplement: Supplementary file 1 [file Image10.jpg]

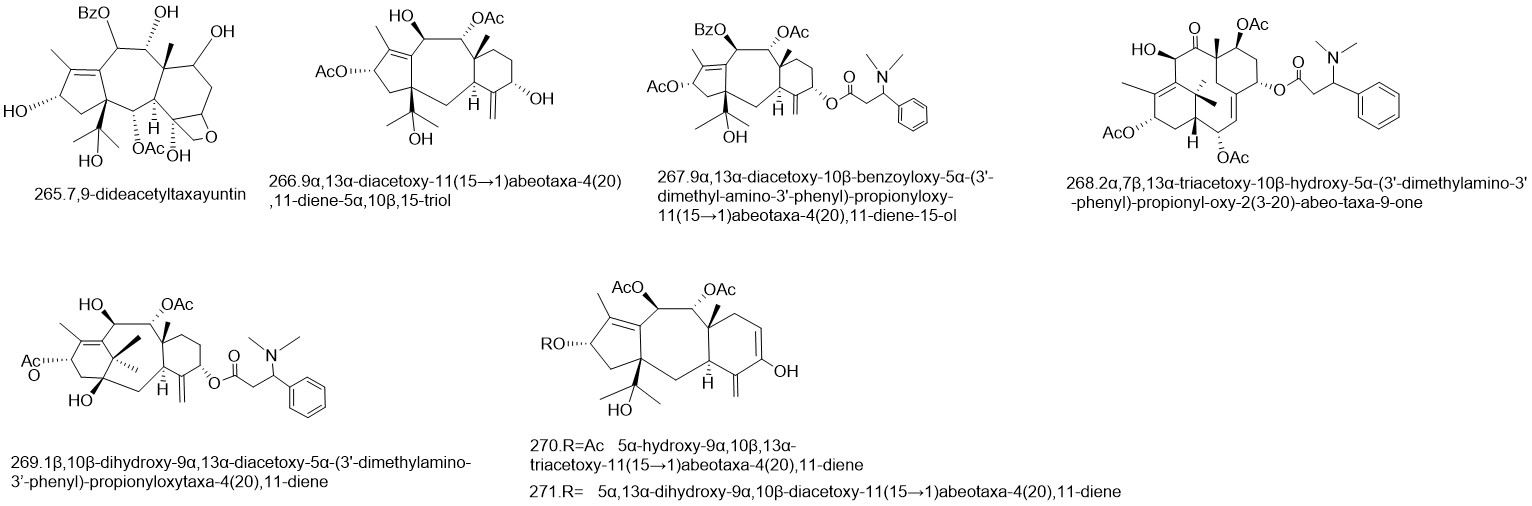

Supplement: Supplementary file 2 [file Image5.jpg]

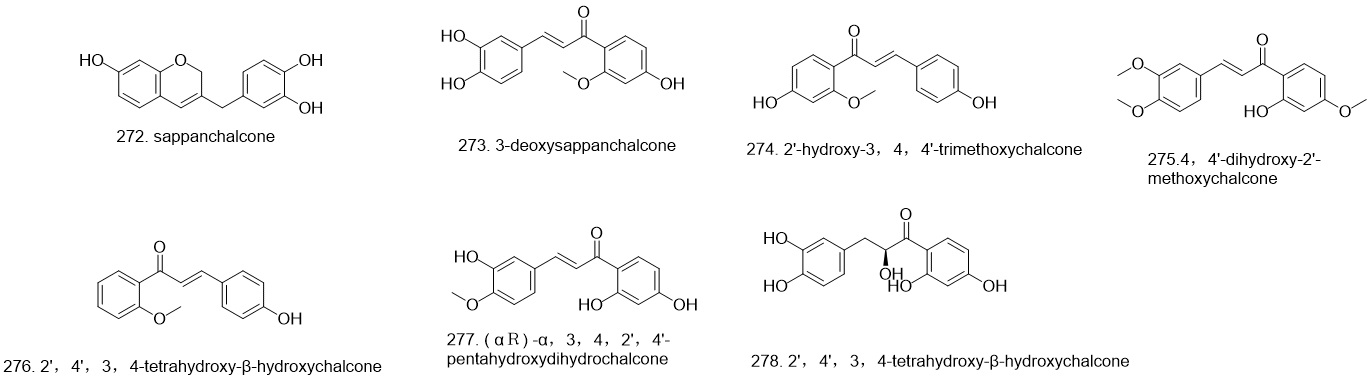

Supplement: Supplementary file 3 [file Image6.jpg]

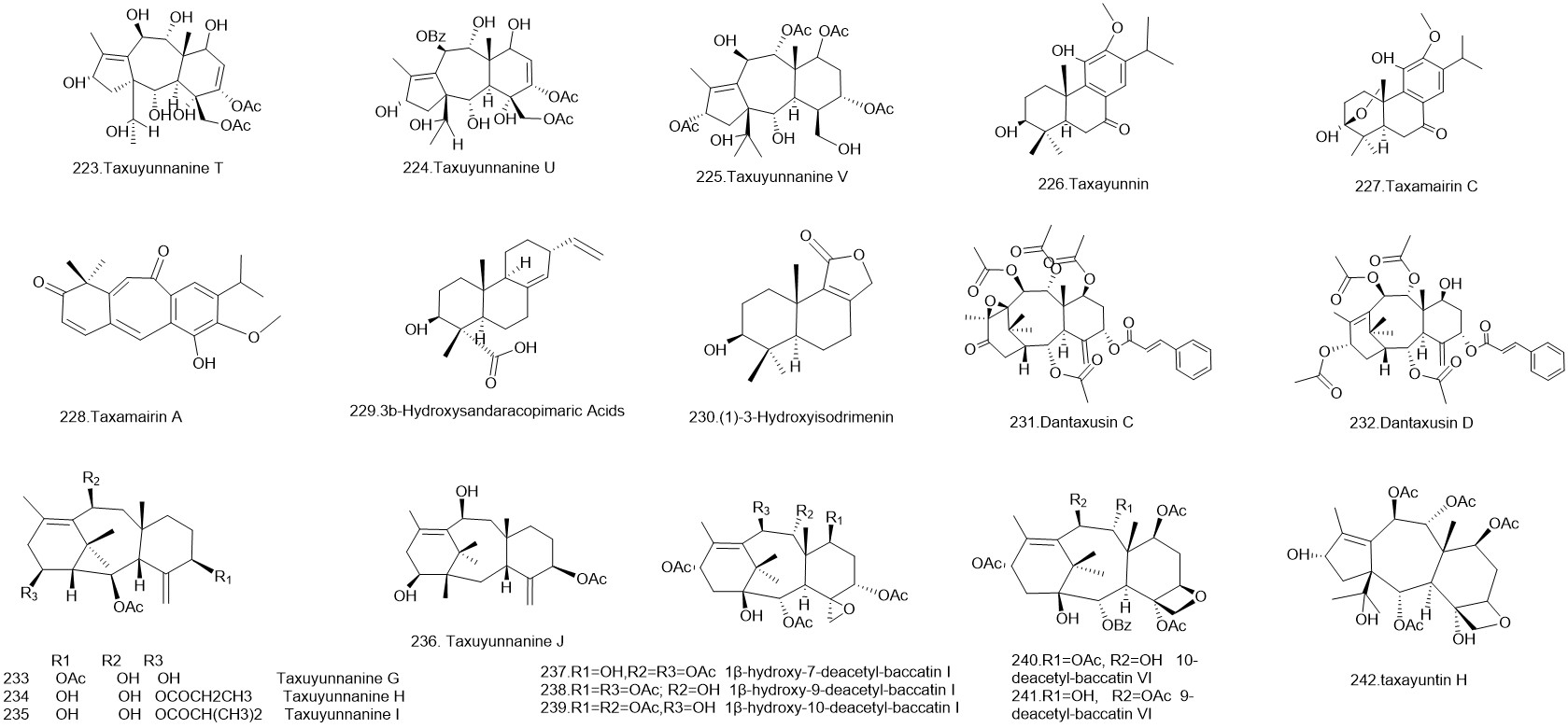

Supplement: Supplementary file 4 [file Image3.jpg]

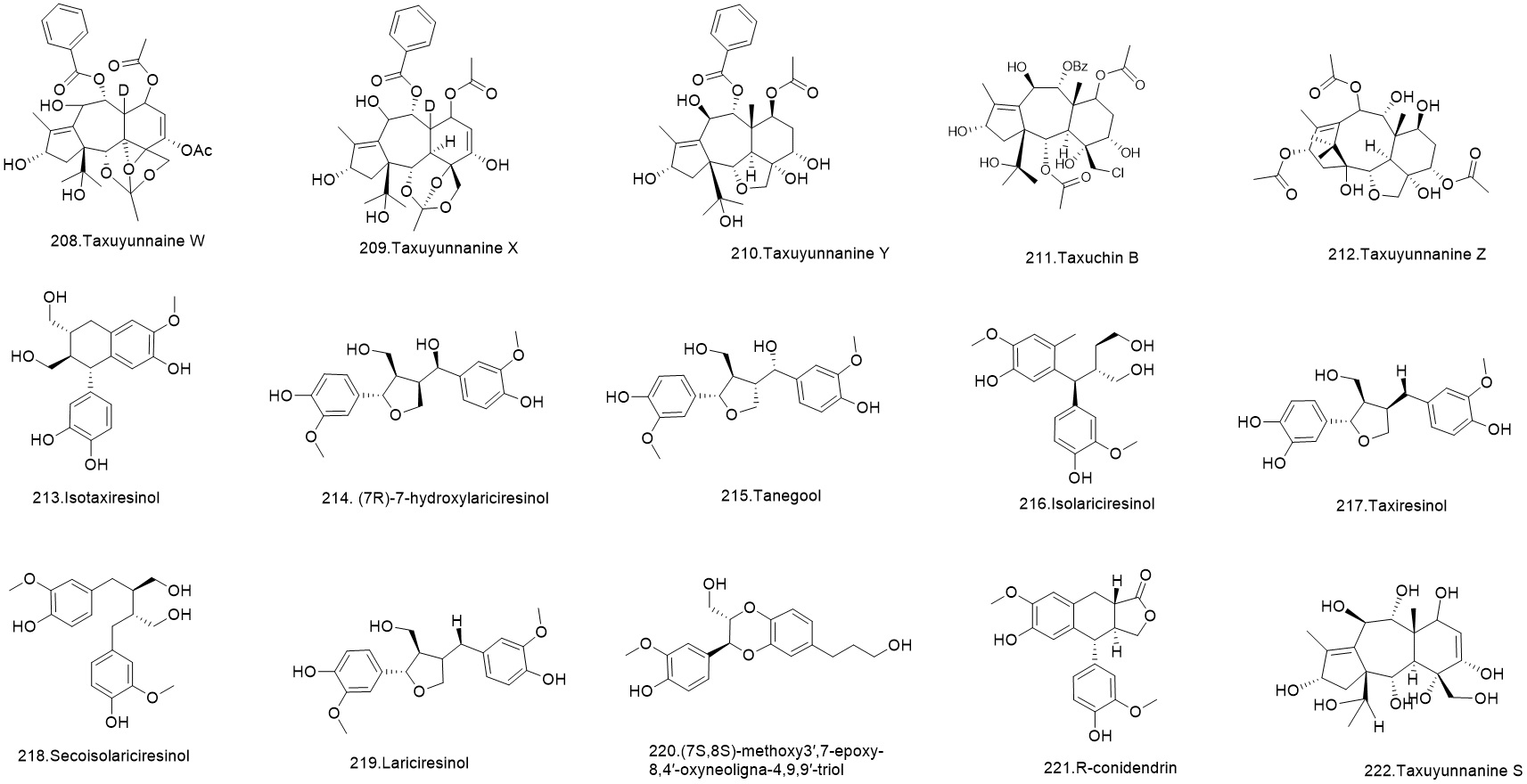

Supplement: Supplementary file 5 [file Image2.jpg]

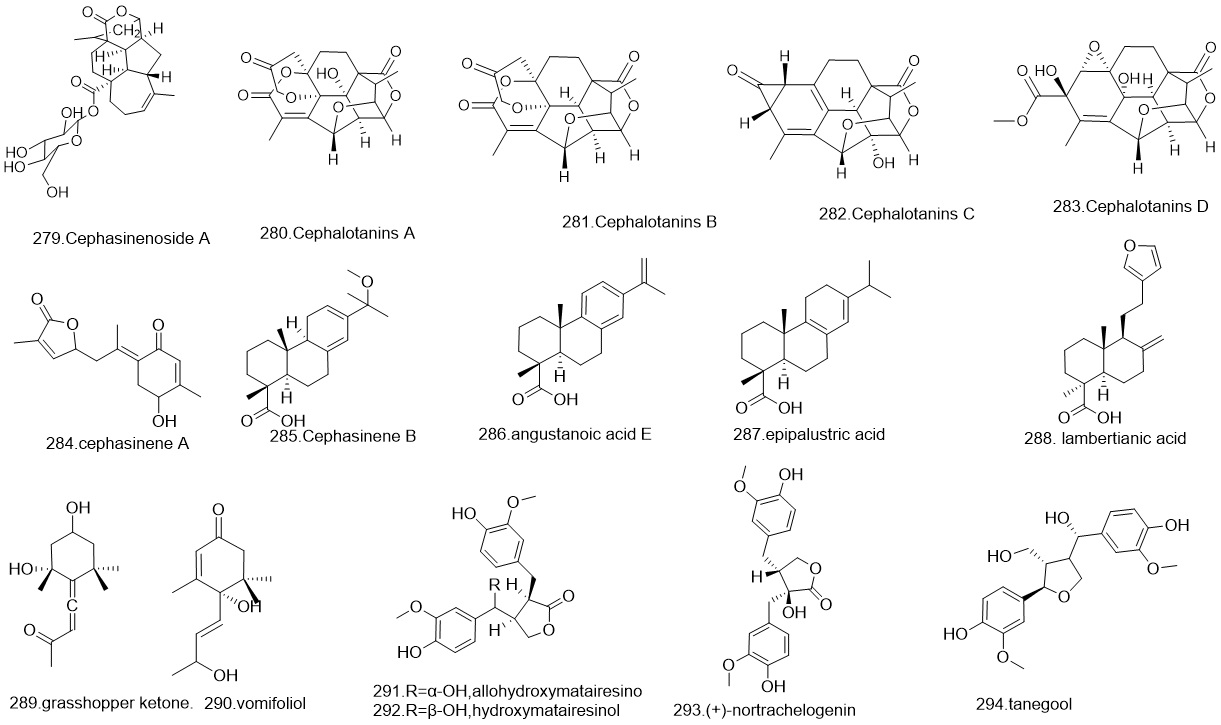

Supplement: Supplementary file 6 [file Image7.jpg]

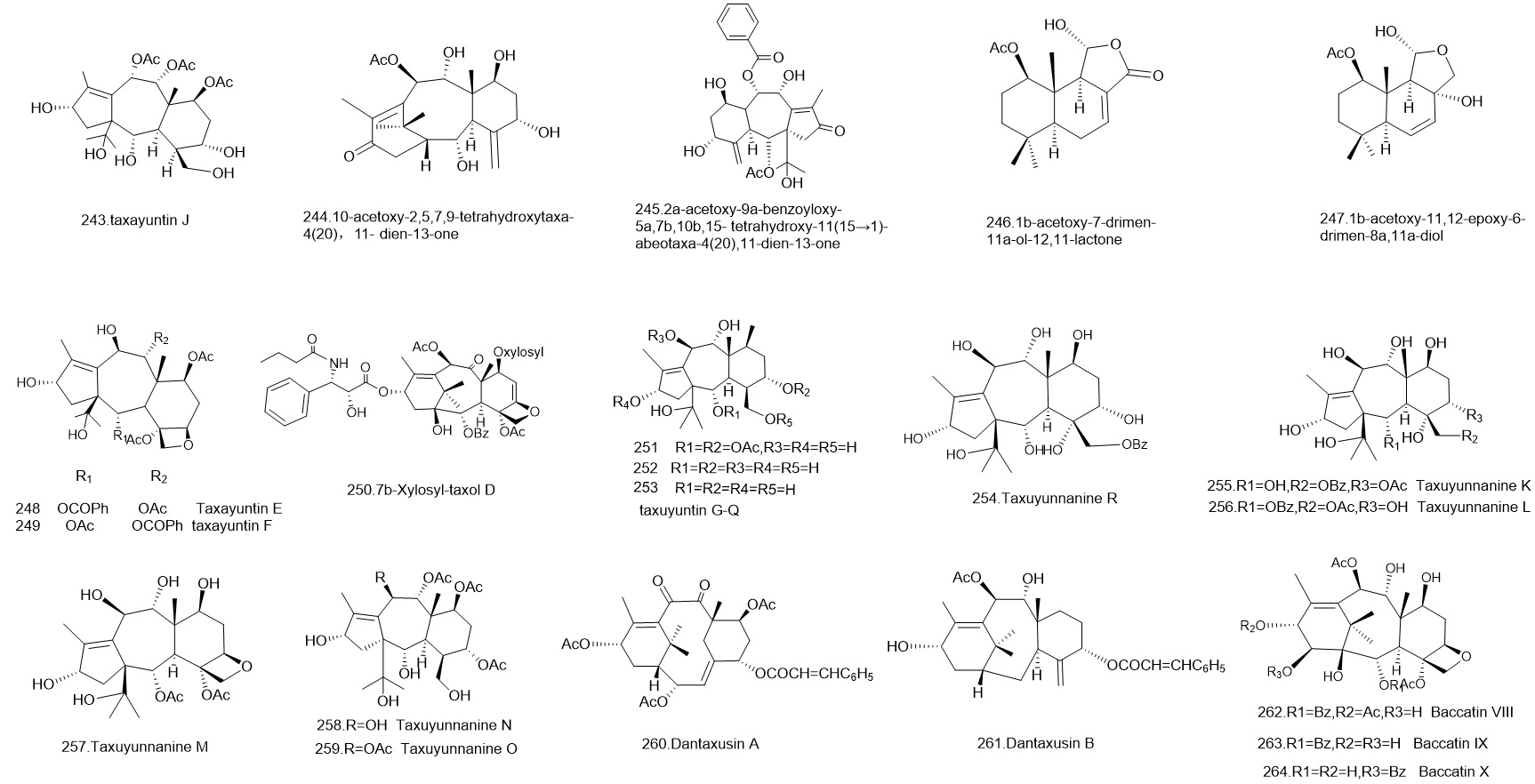

Supplement: Supplementary file 7 [file Image4.jpg]

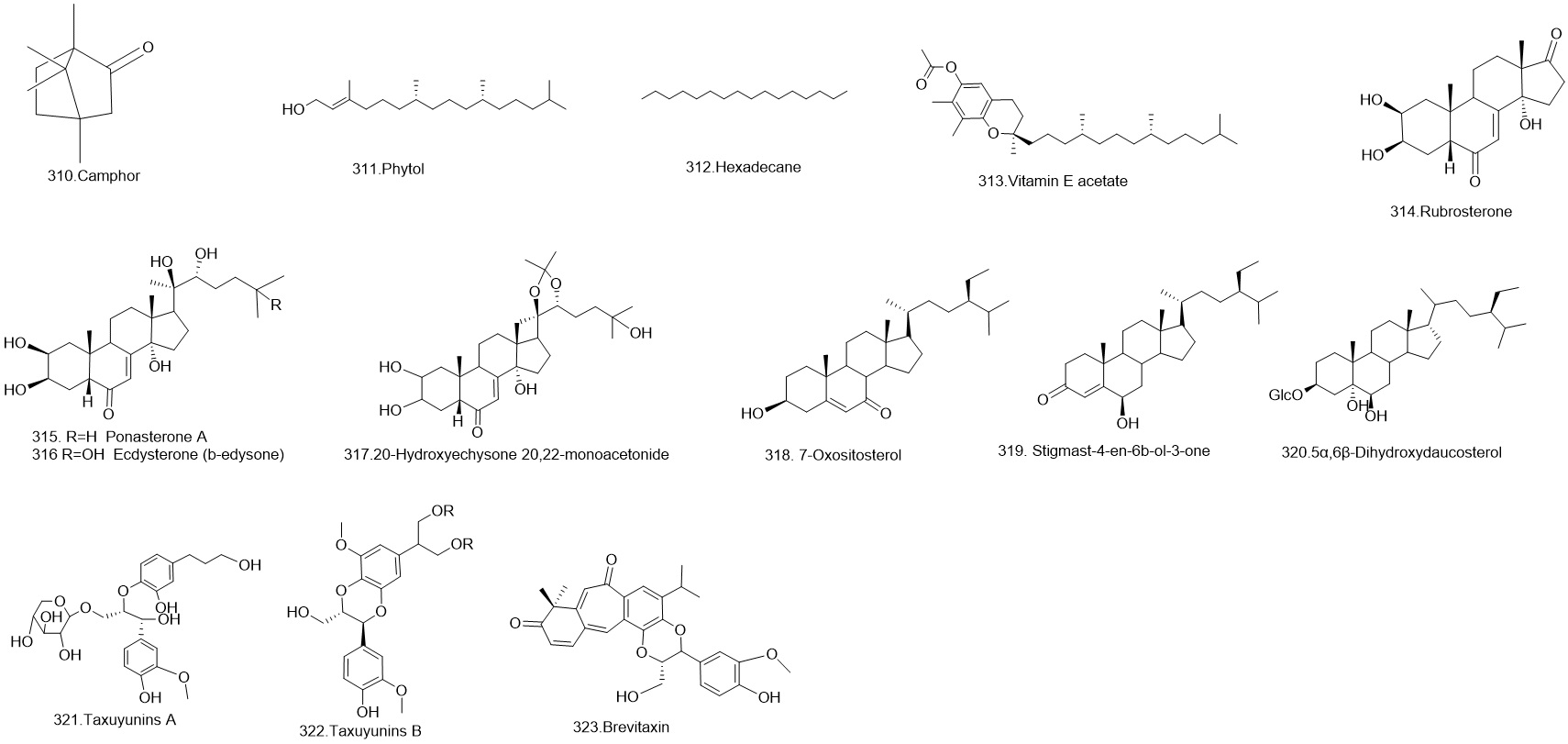

Supplement: Supplementary file 8 [file Image9.jpg]

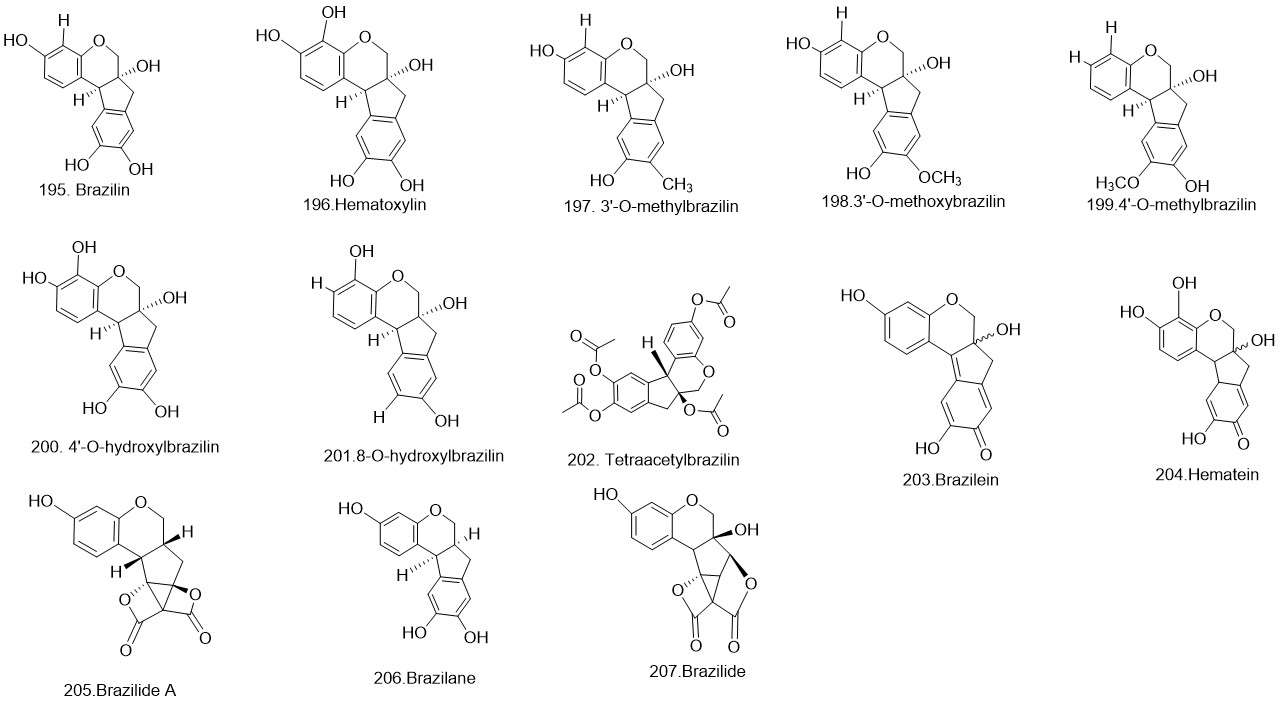

Supplement: Supplementary file 9 [file Image1.jpg]

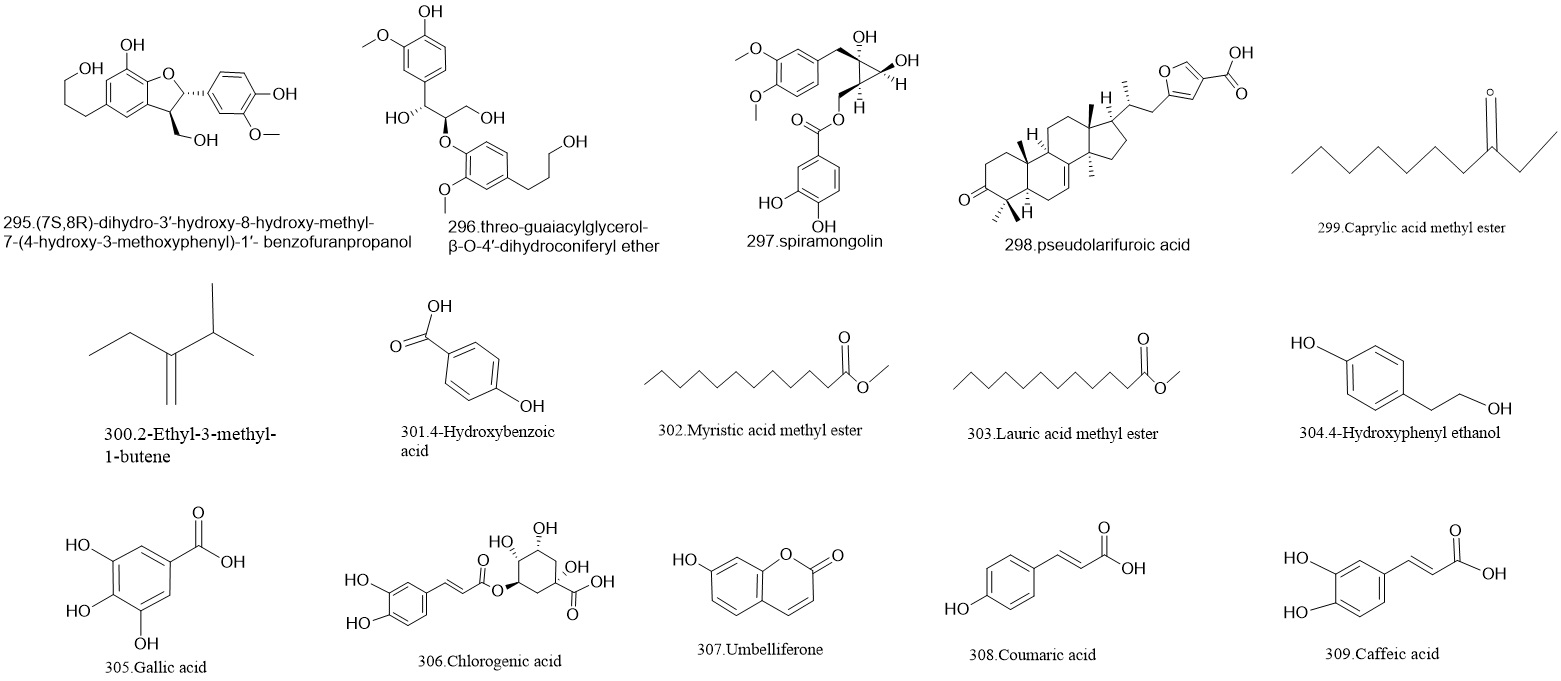

Supplement: Supplementary file 10 [file Image8.jpg]
