# Supplementary material for: A comprehensive review of Shengdeng in Tibetan medicine: textual research, herbal and botanical distribution, traditional uses, phytochemistry, and pharmacology
Source: Front Pharmacol. 2023 Dec 14;14:1303902. doi: 10.3389/fphar.2023.1303902 (PMC10762315; doi:10.3389/fphar.2023.1303902)
Supplement: Supplementary file 11 [file DataSheet1.docx]

## **Table 5** The compound names, compound type and corresponding plant sources.

| NO | Compound name | Type | Source | Reference |
| --- | --- | --- | --- | --- |
|  | Kaempferol | Flavonoids | *X. sorbifolium、R. gilgitica* | Ni and zhang (2009); Yang et al. (2020) |
|  | Quercetin | Flavonoids | *X. sorbifolium、R. gilgitica* | Ni and zhang (2009); Yang et al. (2020) |
|  | Myricetin | Flavonoids | *X. sorbifolium* | Ni and zhang (2009); Yang et al. (2020) |
|  | Naringenin | Flavonoids | *X. sorbifolium、R. gilgitica* | Ni and zhang (2009); Yang et al. (2020) |
|  | Eriodictyol | Flavonoids | *X. sorbifolium* | Ni and zhang (2009); Yang et al. (2020) |
|  | Dihydrokaempferol | Flavonoids | *X. sorbifolium* | Ni and zhang (2009); Yang et al. (2020) |
|  | Taxifolin | Flavonoids | *X. sorbifolium* | Ni and zhang (2009); Yang et al. (2020) |
|  | Dihydromyricetin | Flavonoids | *X. sorbifolium* | Ni and zhang (2009); Yang et al. (2020) |
|  | 5,7,3′, 4′,5′-pentahydroxyflavanone | Flavonoids | *X. sorbifolium* | Ni and zhang (2009); Yang et al. (2020) |
|  | (-)-epigallocatechin | Flavonoids | *X. sorbifolium* | Ni and zhang (2009); Yang et al. (2020) |
|  | L-Epicatechin | Flavonoids | *X. sorbifolium* | Ni and zhang (2009); Yang et al. (2020) |
|  | Procyanidin A2 | Flavonoids | *X. sorbifolium* | Ni and zhang (2009); Yang et al. (2020) |
|  | 3-*O*-methylquercetin | Flavonoids | *X. sorbifolium* | Ni and zhang (2009); Yang et al. (2020) |
|  | Isoquercitrin | Flavonoids | *X. sorbifolium* | Ni and zhang (2009) |
|  | Isomyricitrin | Flavonoids | *X. sorbifolium* | Ni and zhang (2009) |
|  | Myricetin-3-*O*-rutinoside | Flavonoids | *X. sorbifolium* | Ni and zhang (2009) |
|  | Rutinum | Flavonoids | *X. sorbifolium* | Ni and zhang (2009) |
|  | Rhamnocitrin | Flavonoids | *X. sorbifolium* | Ni and zhang (2009) |
|  | Chrysoeriol | Flavonoids | *X. sorbifolium* | Ni and zhang (2009) |
|  | Luteolin | Flavonoids | *X. sorbifolium* | Wan et al. (2015) |
|  | (−)-epiafzelechin | Flavonoids | *X. sorbifolium* | Ma et al. (2004) |
|  | Apigenin-5-*O*-*β*-D-glucoside | Flavonoids | *C. sinensis* | Jiang et al. (2013) |
|  | Apigenin-7-*O*-*β*-D-glucoside | Flavonoids | *C. sinensis* | Jiang et al. (2013) |
|  | Isoquercitroside | Flavonoids | *C. sinensis* | Jiang et al. (2013) |
|  | Scutellarein-5-*O*-[*α*-L-rhamnopyranosyl-(1 →4)-  *β*-D-6-*O*-acetylglucopyranoside] | Flavonoids | *C. sinensis* | Li et al. (2007a) |
|  | Apigenin-5-*O*-[*α*-L-rhamnopyranosyl-(1→ 2)-*β*-D-3,6-di-*O*-acetylglucopyranoside] | Flavonoids | *C. sinensis* | Li et al. (2007a) |
|  | (+)-Catechin | Flavonoids | *A. catechu* | Adhikari et al., (2021) |
|  | (–)-Epicatechin | Flavonoids | *A. catechu* | Adhikari et al., (2021) |
|  | Epiafzelechin | Flavonoids | *A. catechu* | Adhikari et al., (2021) |
|  | Afzelechin | Flavonoids | *A. catechu* | Adhikari et al., (2021) |
|  | Mesquitol | Flavonoids | *A. catechu* | Adhikari et al., (2021) |
|  | Ophioglonin | Flavonoids | *A. catechu* | Adhikari et al., (2021) |
|  | Aromadendrin | Flavonoids | *A. catechu、*  *R. gilgitica* | Adhikari et al., (2021) |
|  | Kaempferol | Flavonoids | *A. catechu* | Adhikari et al., (2021) |
|  | Baicalin | Flavonoids | *A. catechu* | Adhikari et al., (2021) |
|  | Baicalein | Flavonoids | *A. catechu* | Adhikari et al., (2021) |
|  | Quercetin | Flavonoids | *A. catechu* | Adhikari et al., (2021) |
|  | Quercetin 3-methyl ether | Flavonoids | *A. catechu* | Hong et al. (2015) Adhikari et al., (2021) |
|  | Caryatin | Flavonoids | *A. catechu* | Hong et al. (2015), Adhikari et al., (2021) |
|  | Ellagic acid | Flavonoids | *A. catechu* | Hong et al. (2015) Adhikari et al., (2021) |
|  | 5-Hydroxy-2-[2-(4-hydroxyphenyl)acetyl]-3-methoxylbenzoic acid | Flavonoids | *A. catechu* | Li et al. (2011)  Adhikari et al., (2021) |
|  | (2*S*,3*S*)-3,7,8,3´,4´-pentahydroxyflavane | Flavonoids | *A. catechu* | Li et al. (2011) Adhikari et al., (2021) |
|  | Rhamnetin | Flavonoids | *A. catechu* | Li et al. (2011), Adhikari et al., (2021) |
| 1. , | 3,3´,5,5´,7-pentahydroxyflavane  **(+)-** Dihydrorobinetin | Flavonoids | *A. catechu* | Li et al. (2011), Adhikari et al., (2021) |
|  | Fisetinidol | Flavonoids | *A. catechu* | Li et al. (2011), Adhikari et al., (2021) |
|  | Rutin | Flavonoids | *A. catechu* | Negi and Dave (2010) |
|  | Gallocatechin | Flavonoids | *A. catechu* | Adhikari et al., (2021) |
|  | Epigallocatechin | Flavonoids | *A. catechu* | Adhikari et al., (2021) |
|  | Procyanidin B1 | Flavonoids | *A. catechu* | Adhikari et al., (2021) |
|  | Procyanidin B3 | Flavonoids | *A. catechu* | Adhikari et al., (2021) |
|  | Emodin | Flavonoids | *A. catechu* | Adhikari et al., (2021) |
|  | Maclurin | Flavonoids | *A. catechu* | Adhikari et al., (2021) |
|  | Irisflorentin | Flavonoids | *A. catechu* | Adhikari et al., (2021) |
|  | Naringenin | Flavonoids | *A. catechu* | Adhikari et al., (2021) |
|  | Isoquercetin | Flavonoids | *A. catechu* | Adhikari et al., (2021) |
|  | Diosmetin | Flavonoids | *A. catechu* | Adhikari et al., (2021) |
|  | Chrysin | Flavonoids | *A. catechu* | Adhikari et al., (2021) |
|  | Myricetin | Flavonoids | *A. catechu* | Adhikari et al., (2021) |
|  | Avicularin | Flavonoids | *A. catechu* | Adhikari et al., (2021) |
|  | Prodelphinidin B | Flavonoids | *A. catechu* | Adhikari et al., (2021) |
|  | Prodelphinidin B3 | Flavonoids | *A. catechu* | Adhikari et al., (2021) |
|  | Taxifolin | Flavonoids | *A. catechu、R. gilgitica* | Adhikari et al., (2021) |
|  | Acacetin | Flavonoids | *A. catechu* | Adhikari et al., (2021) |
|  | Aciculatinone | Flavonoids | *A. catechu* | Adhikari et al., (2021) |
|  | Gossypin | Flavonoids | *A. catechu* | Adhikari et al., (2021) |
|  | Pterocarpin | Flavonoids | *A. catechu* | Adhikari et al., (2021) |
|  | Isorhamnetin | Flavonoids | *A. catechu* | Adhikari et al., (2021) |
|  | Trihydroxy dimethoxyflavone | Flavonoids | *A. catechu* | Adhikari et al., (2021) |
|  | Kaempferol-7-*O*-*β*-D-glucoside | Flavonoids | *R. gilgitica* | Pan et al. (1998) |
|  | Sappanone A | Flavonoids | *C. sappan* | Tang et al. (2012) |
|  | 3-deoxysappanone B | Flavonoids | *C. sappan* | Chen et al. (2008) |
|  | 3'-deoxysappanone B | Flavonoids | *C. sappan* | Wang et al. (2003) |
|  | Sappanone B | Flavonoids | *C. sappan* | Chen et al. (2008) |
|  | Tectorigenin | Flavonoids | *C. sappan* | Zhao et al. (2010) |
|  | 8-methoxybonducellin | Flavonoids | *C. sappan* | Chen et al. (2012) |
|  | 3，7-hydroxy-3-( 3'，4'-hydroxybenzyl) -  benzodihydro-4-one | Flavonoids | *C. sappan* | Zhou et al. (2017) |
|  | 3，9-dihydroxy-8-methoxy-dibenzo ［b，d］ pyran-6-one | Flavonoids | *C. sappan* | Wang and Liang (2016) |
|  | 3'，4'，7-trihydroxyisoflavanone | Flavonoids | *C. sappan* | Wang (2006) |
|  | ( ±) -7，3'，4'-trihydroxyflavanone | Flavonoids | *C. sappan* | Wang (2006) |
|  | 3'，4'，7-trihydroxyisoflavone | Flavonoids | *C. sappan* | Wang (2006) |
|  | 3，7，3'4'-tetrahydroxyflavanone fustin | Flavonoids | *C. sappan* | Wang (2006) |
|  | 3，7-dihydroxy-chromen-4-one | Flavonoids | *C. sappan* | Wang and Liang (2016) |
|  | 5，7-dihydroxy-4'-methoxyisoflavone | Flavonoids | *C. sappan* | Wang et al. (2010) |
|  | 7-hydroxy-3-( 3'，4'-dihydroxy-  benzylidene) -chroman-4-one | Flavonoids | *C. sappan* | Wang et al. (2003) |
|  | 3，7-dihydroxy-3-( 4'-hydroxybenzyl) -chroman-4-one | Flavonoids | *C. sappan* | Namikoshi et al. (1987) |
|  | 7-hydroxy-8-methoxy-3-( 4'-  methoxybenzylidene) -chroman-4-one | Flavonoids | *C. sappan* | Wang et al. (2003) |
|  | 7-hydroxy-3-( 4'-hydroxybenzylidene) -  chroman-4-one | Flavonoids | *C. sappan* | Wang et al. (2003) |
|  | 3-( 3'，4'-dihydroxybenzyl) -7-  hydroxychroma-4-one | Flavonoids | *C. sappan* | Wang (2006) |
|  | 3，7-dihydroxy-chroman-4-one | Flavonoids | *C. sappan* | Shu (2007) |
|  | 3，8，9-trihydroxy-6H-benzo  ［c］ chromen-6-one | Flavonoids | *C. sappan* | Wang and Liang (2016) |
|  | ( *E*) -3-( 3，4-dihydroxybenzylidene) -7-  hydroxychroman-4-one | Flavonoids | *C. sappan* | Chen (2008) |
|  | 3*R*， 4*S* ) -3-( 3'， 4'-hydroxybenzyl )  -3， 4-dihydro-2″， 3″- dimethyl-3H-  ［1，3］ dioxolo ［4，5-c］ -chromen-7-ol | Flavonoids | *C. sappan* | Sheng (2016) |
|  | 3，9-dihydroxy-8-methoxy-dibenzo ［b，d］ pyran-6-one | Flavonoids | *C. sappan* | Wang and Liang (2016) |
|  | 3，4，7-trihydroxy-3-( 4'-hydroxybenzyl) -  chroman-4-one | Flavonoids | *C. sappan* | Namikoshi et al. (1987) |
|  | 7，4'-dimethoxyhomoisoflavanone | Flavonoids | *C. sappan* | Cai (2012) |
|  | 7，3'，5'-trihydroxydihydroflavone | Flavonoids | *C. sappan* | Wang and Liang (2016) |
|  | 5，7-dihydroxy-4'-methoxydihydroisoflavone | Flavonoids | *C. sappan* | Wang et al. (2010) |
|  | 7，3'，4'-trihydroxydihydroisoflavone | Flavonoids | *C. sappan* | Wang et al. (2010) |
|  | 7，3'，4'-trihydroxydihydroflavone | Flavonoids | *C. sappan* | Wang et al. (2010) |
|  | 7，3'，4'-trihydroxyisoflavone | Flavonoids | *C. sappan* | Wang et al. (2010) |
|  | 3，7，3'，4'-tetrahydroxy-4-dimethoxy-homoisoflavans | Flavonoids | *C. sappan* | Wang and Liang (2016) |
|  | Epi-Sappanol | Flavonoids | *C. sappan* | Wang et al. (2003) |
|  | 3'-deoxysappanol | Flavonoids | *C. sappan* | Wang and Liang (2016) |
|  | 3'-*O*-methylsappanol | Flavonoids | *C. sappan* | Wang et al, (2013) |
|  | Episappanol | Flavonoids | *C. sappan* | Wang et al. (2003) |
|  | 3'-*O*-methylepisappanol | Flavonoids | *C. sappan* | Wang et al. (2003) |
|  | 4-*O*-methylepisappanol | Flavonoids | *C. sappan* | Wang et al. (2003) |
|  | 3'-deoxy-4-*O*-methylepisappanol | Flavonoids | *C. sappan* | Wang and Liang (2016) |
|  | 4-*O*-methylsappanol | Flavonoids | *C. sappan* | Wang et al. (2003) |
|  | 13'-deoxy-4-*O*-methylsappanol | Flavonoids | *C. sappan* | Zhao et al. (2014) |
|  | ( － ) -5-*O*-methyllatifolin | Homoisoflavones | *C. sappan* | Wang et al. (2013) |
|  | ( － ) -dalbergiphenol | Homoisoflavones | *C. sappan* | Wang et al. (2013) |
|  | ( － ) -latifolin | Homoisoflavones | *C. sappan* | Wang et al. (2013) |
|  | Neoprotosappanin | Homoisoflavones | *C. sappan* | Nguyen et al.(2005) |
|  | Neosappanone A | Homoisoflavones | *C. sappan* | Shu (2007) |
|  | Neocaesalpin A | Homoisoflavones | *C. sappan* | Shu (2007) |
|  | Neocaesalpin B | Homoisoflavones | *C. sappan* | Shu (2007) |
|  | 3，8-dihydroxy-4，10-dimethoxy-7-oxo-［2］ benzopyrano  ［4，3-b］ benzopyran | Homoisoflavones | *C. sappan* | Shu (2007) |
|  | (*S*) -7-( dihydroxymethyl) -7，8-dihydro-6H-dibenzo ［b，  d］ oxocine-3，7，10，11-tetraol | Homoisoflavones | *C. sappan* | Zhou (2017) |
|  | ( 7，3'，4'-trihydroxy-3-benzyl-2H-chromene) | Homoisoflavones | *C. sappan* | Zhao et al. (2014), Wan et al (2013) |
|  | 21,22-di-*O*-angeloyl-R1-barrigenol | Triterpenoids | *X. sorbifolium* | Chen et al. (1984), Wan et al (2013) |
|  | 21,22-di-*O*-angeloyl-24-hydroxy-R1-barrigenol | Triterpenoids | *X. sorbifolium* | Li (2006), Wan et al (2013) |
|  | 21-*O*-angeloyl-R1-barrigenol | Triterpenoids | *X. sorbifolium* | Li (2004), Wan et al (2013) |
|  | 22-*O*-angeloyl-R1-barrigenol | Triterpenoids | *X. sorbifolium* | Buren (2007), Wan et al (2013) |
|  | R1-barrigenol | Triterpenoids | *X. sorbifolium* | Li (2006), Wan et al (2013) |
|  | 3-*O*-(3-*O*-*α*-L-arabinofuranosyl-2-*O*-*β*-D-galactopyranosyl)- *β*-*O*-glucopyranosyl-21, 22-di-*O*-angeloyl-R1-barrigenol | Triterpenoids | *X. sorbifolium* | Li (2006), Wan et al (2013) |
|  | 22-*O*-angeloyl-21-*O*-epoxyangeloyl-barringtogenol C | Triterpenoids | *X. sorbifolium* | Li (2006), Wan et al (2013) |
|  | 21,22-di-*O*-angeloyl-barringtogenol C | Triterpenoids | *X. sorbifolium* | Li (2006), Wan et al (2013) |
|  | Barringtogenol C | Triterpenoids | *X. sorbifolium* | Li (2006), Wan et al (2013) |
|  | 16-deoxybarringtogenol C | Triterpenoids | *X. sorbifolium* | Li (2006), Wan et al (2013) |
|  | 28-*O*-*β*-D-glucopyranosyl-16-deoxybarringtogenol | Triterpenoids | *X. sorbifolium* | Li (2006), Wan et al (2013) |
|  | 16-*O*-acetyl-21-*O*-(4-*O*-angeloyl-*α*-L-rhamnopytanosyl)- barringtogenol C | Triterpenoids | *X. sorbifolium* | Li (2006), Wan et al (2013) |
|  | 3-*O*-*β*-D-glucopyranosyl (1→6)-*β*-D-glucopyranosyl-28- O-*β*-D-glucopyranosyl (1→6)[*α*-L-rhamnopytanosyl (1→ 2)]-*β*-D-glucopyranosyl-16-deoxybarringtogenol C | Triterpenoids | *X. sorbifolium* | Li et al.(2006) |
|  | 3-*O*-[*β*-D-glucopyranosyl (1→6)] (3′-*O*-angeloyl)- *β*-D-glucopyranosyl-28-*O*-[*α*-L-rhamnopytanosyl (1→2)]-*β*-D-glucopyranosyl-16- deoxybarringtogenol C | Triterpenoids | *X. sorbifolium* | Li et al.(2008) |
|  | 3-*O*-*β*-D-glucopyranosyl-16-deoxybarringtogenol C | Triterpenoids | *X. sorbifolium* | Wang et al. (2011) |
|  | 16-*O*-acetyl-21-*O*-(3′, 4′-di-*O*-angeloyl)-*β*-D-fucopyranosyl theasapogenol B | Triterpenoids | *X. sorbifolium* | Li et al. (2007a) |
|  | 22-*O*-acetyl-21-*O*-(4′-*O*-angeloyl)-*β*-D-fucopyranosyl theasapogenol B | Triterpenoids | *X. sorbifolium* | Li et al.(2006) |
|  | 28-*O*-*β*-D-glucopyranosyl-21-*O*-angeloyl-R1-barrigenol | Triterpenoids | *X. sorbifolium* | Wang et al. (2011) |
|  | 21-*O*-angeloyl-24-hydroxy-R1-barrigenol | Triterpenoids | *X. sorbifolium* | Li and Li (2008) |
|  | 3-*O*-(3-*O*-*α*-L-arabinofuranosyl-2-*O*-*β*-D-galactopyranosyl)-(6-*O*-methyl)-*β*-D-glucuronopyranosyl-21, 22-di-*O*-angeloyl-R1-barrigenol | Triterpenoids | *X. sorbifolium* | Wang et al. (2011) |
|  | 3-*O*-[*β*-D-galactopyranosyl(1→2)-*α*-L-arabinofuranosyl (1→3)-*β*-D-methyl glucuronic acid 21-*O*-(3, 4-diangeloyl)-*α*-L-rhamnose-3*β*, 16*α*, 21*β*, 22*α*, 28*β*-pentahydroxyl-22-acetoxy-olean12-ene | Triterpenoids | *X. sorbifolium* | Guo et al.(2009) |
|  | 3-*O*-*β*-D-galactopyranosyl-(1→6)-(2-*O*-angeloyl)- *β*-D-glucopyranosyl saniculagenic C-28-*O*-*α*-L-rhamnopytanosyl-(1→2)-*β*-D-glucopyranoside | Triterpenoids | *X. sorbifolium* | Fu et al. (2010) |
|  | Xanifolia Y0 | Triterpenoids | *X. sorbifolium* | Chan et al. (2008) |
|  | Xanifolia Y2 | Triterpenoids | *X. sorbifolium* | Chan et al. (2008) |
|  | Xanifolia Y3 | Triterpenoids | *X. sorbifolium* | Chan et al. (2008) |
|  | Xanifolia Y7 | Triterpenoids | *X. sorbifolium* | Chan et al. (2008) |
|  | Sorbifoliumside G | Triterpenoids | *X. sorbifolium* | Yu et al. (2012a) |
|  | Sorbifoliumside H | Triterpenoids | *X. sorbifolium* | Yu et al. (2012a) |
|  | Sorbifoliumside I | Triterpenoids | *X. sorbifolium* | Yu et al. (2012a) |
|  | Sorbifoliumside J | Triterpenoids | *X. sorbifolium* | Yu et al. (2012a) |
|  | 3-*O*-*β*-D-glucopyranosyl-28-*O*-[*β*-D-glucopyranosyl (1→2)]-*β*-D-glucopyranosyl-21*β*, 22*α*- dihydroxyl-olean-12-ene | Triterpenoids | *X. sorbifolium* | Cui et al. (2012) |
|  | Sorbifoliumsides A | Triterpenoids | *X. sorbifolium* | Yu et al. (2012a) |
|  | Sorbifoliumsides B | Triterpenoids | *X. sorbifolium* | Yu et al. (2012a) |
|  | Sorbifoliumsides C | Triterpenoids | *X. sorbifolium* | Yu et al. (2012a) |
|  | Sorbifoliumsides D | Triterpenoids | *X. sorbifolium* | Yu et al. (2012a) |
|  | Sorbifoliumsides E | Triterpenoids | *X. sorbifolium* | Yu et al. (2012a) |
|  | Sorbifoliumsides F | Triterpenoids | *X. sorbifolium* | Yu et al. (2012a) |
|  | Bunkankasaponin F | Triterpenoids | *X. sorbifolium* | Yu et al. (2012a) |
|  | 3-*O*-*β*-D-glucopyranosyl (1→6)-[angeloyl (1→ 2)]-*β*-D-glucopyranosyl-28-*O*-*α*-L-rhamnopyranosyl (1→2)-[*β*-D-glucopyranosyl (1→6)]- *β*-D-glucopyranosyl-21*β*, 22*α*-dihydroxylolean-12-ene | Triterpenoids | *X. sorbifolium* | Cui et al. (2012) |
|  | 3-*O*-*β*-D-glucopyranosyl,28-*O*-[*α*-L-rhamnosyl (1→2)]-*β*-D- glucopyranosyl-16- deoxybarringtogenol C | Triterpenoids | *X. sorbifolium* | Li et al.(2008) |
|  | 3-*O*-[*β*-D-galactopyranosyl (1→2)]-*α*-L-arabinofuranosyl (1→3)-*β*-D-methyl glucuronic acid21, 22-*O*-diangeloyl-3*β*, 15*α*, 16*α*, 21*β*, 22*α*, 28*β*-hexahydroxyl-olean-12-ene | Triterpenoids | *X. sorbifolium* | Guo et al.(2009) |
|  | 22-*O*-acnapoleogenin B | Triterpenoids | *X. sorbifolium* | Chen et al. （1985a) |
|  | Napoleogenin B | Triterpenoids | *X. sorbifolium* | Chen et al. （1985a) |
|  | 21-*O*-(3, 4-di-*O*-angeloyl)-*β*-D-fucopyranosyl theasapogenol B | Triterpenoids | *X. sorbifolium* | Chen et al. （1985a) |
|  | 21-*O*-(4-*O*-acetyl-3-*O*-angeloyl)-*β*-D-fucopyranosyl theasapogenol B | Triterpenoids | *X. sorbifolium* | Chen et al. （1985a) |
|  | 21-*O*-(4-*O*-acetyl-3-*O*-angeloyl)-*β*-D-fucopyranosyl-22-*O*-acetyl protoaescigenin | Triterpenoids | *X. sorbifolium* | Chen et al. （1985a) |
|  | 16-*O*-acetyl-21-*O*-(3, 4-di-*O*-angeloyl)-*β*-Dfucopyranosyl protoaescigen | Triterpenoids | *X. sorbifolium* | Chen et al. （1985a) |
|  | 3-*O*-*β*-D-glucuronopyranoside bunkankasaponin A | Triterpenoids | *X. sorbifolium* | Chen et al. （1985b) |
|  | Bunkankasaponin B | Triterpenoids | *X. sorbifolium* | Chen et al. （1985b) |
|  | Bunkankasaponin C | Triterpenoids | *X. sorbifolium* | Chen et al. （1985b) |
|  | Bunkankasaponin D | Triterpenoids | *X. sorbifolium* | Chen et al. （1985b) |
|  | 3*β*,23-dihydroxy-lup-20(29)-en-28-oic acid-23-caffeate | Triterpenoids | *X. sorbifolium* | Li et al. (2007a) |
|  | 3*β*,23-dihydroxy-lup-20(29)-en-28-oic acid-3-caffeate | Triterpenoids | *X. sorbifolium* | Li and Li (2008) |
|  | Betulin | Triterpenoids | *X. sorbifolium* | Li and Li (2008) |
|  | 23-hydroxybetulinic acid | Triterpenoids | *X. sorbifolium* | Li and Li (2008) |
|  | 3*β*-hydroxytirucalla-7, 24-dien-21-oic acid | Triterpenoids | *X. sorbifolium* | Ma et al. (2000) |
|  | 3*α*, 29-dihydroxytirucalla-7, 24-dien-21-oic acid | Triterpenoids | *X. sorbifolium* | Ma et al. (2000) |
|  | 3*β*, 29-dihydroxytirucalla-7, 24-dien-21-oic acid | Triterpenoids | *X. sorbifolium* | Ma et al. (2000) |
|  | 3-oxotriucalla-7, 24-dien-21-oic acid | Triterpenoids | *X. sorbifolium* | Ma et al. (2000) |
|  | 29-hydroxy-3-oxotirucalla-7, 24-dien-21-oic acid | Triterpenoids | *X. sorbifolium* | Ma et al. (2000) |
|  | 29-*O*-acetyl-3-oxotirucalla-7, 24-dien-21-oic acid | Triterpenoids | *X. sorbifolium* | Ma et al. (2000) |
|  | Barrigtogenol C | Triterpenoids | *X. sorbifolium* | Li (2006) |
|  | 16-deoxybarrigtogenol C | Triterpenoids | *X. sorbifolium* | Li (2006) |
|  | Oleanolic acid | Triterpenoids | *X. sorbifolium* | Qi (2018) |
|  | Protoaescigenin | Triterpenoids | *X. sorbifolium* | Qi (2018) |
|  | Protosappanin A | Protosappanins | *C. sappan* | Wang and Liang (2016) |
|  | Protosappanin B | Protosappanins | *C. sappan* | Li et al. (2012) |
|  | Protosappanin C | Protosappanins | *C. sappan* | Shu, (2007) |
|  | Isoprotosappanin B | Protosappanins | *C. sappan* | Li et al. (2012) |
|  | 10-*O*-methylprotosappanin B | Protosappanins | *C. sappan* | Li et al. (2012) |
|  | 10-*O*-methylisoprotosappanin B | Protosappanins | *C. sappan* | Li et al. (2012) |
|  | Protosappanin D | Protosappanins | *C. sappan* | Wang et al. (2003) |
|  | Protosappanin E1 | Protosappanins | *C. sappan* | Li et al. (2012) |
|  | Protosappanin E2 | Protosappanins | *C. sappan* | Li et al. (2012) |
|  | Brazilin | Brazilin | *C. sappan* | Zhao et al. (2014) |
|  | Hematoxylin | Brazilin | *C. sappan* | Cooksey (2010) |
|  | 3'-*O*-methylbrazilin | Brazilin | *C. sappan* | Wang et al. (2003) |
|  | 3'-*O*-methoxybrazilin | Brazilin | *C. sappan* | Wang et al. (2013) |
|  | 4'-*O*-methylbrazilin | Brazilin | *C. sappan* | Wang et al. (2003) |
|  | 4'-*O*-hydroxylbrazilin | Brazilin | *C. sappan* | Wang et al. (2003) |
|  | 8-*O*-hydroxylbrazilin | Brazilin | *C. sappan* | Wang et al. (2003) |
|  | Tetraacetylbrazilin | Brazilin | *C. sappan* | Xu (1994) |
|  | Brazilein | Brazilin | *C. sappan* | Shu et al. (2007) |
|  | Hematein | Brazilin | *C. sappan* | Hung et al. (2009) |
|  | Brazilide A | Brazilin | *C. sappan* | Shu, (2007) |
|  | Brazilane | Brazilin | *C. sappan* | Kim and Kim (2018) |
|  | Brazilide | Brazilin | *C. sappan* | Shu et al. (2007) |
|  | Taxuyunnaine W | Taxane | *T. yunnanensis* | Li et al. (2003a) |
|  | Taxuyunnanine X | Taxane | *T. yunnanensis* | Li et al. (2003a) |
|  | Taxuyunnanine Y | Taxane | *T. yunnanensis* | Li et al. (2003a) |
|  | Taxuchin B | Taxane | *T. yunnanensis* | Li et al. (2003a) |
|  | Taxuyunnanine Z | Taxane | *T. yunnanensis* | Li et al. (2003a) |
|  | Isotaxiresinol | Taxane | *T. yunnanensis* | Tezuka et al. (2011) |
|  | (7*R*)-7-hydroxylariciresinol | Taxane | *T. yunnanensis* | Tezuka et al. (2011) |
|  | Tanegool | Taxane | *T. yunnanensis* | Tezuka et al. (2011) |
|  | Isolariciresinol | Taxane | *T. yunnanensis* | Tezuka et al. (2011) |
|  | Taxiresinol | Taxane | *T. yunnanensis* | Tezuka et al. (2011) |
|  | Secoisolariciresinol | Taxane | *T. yunnanensis* | Tezuka et al. (2011) |
|  | Lariciresinol | Taxane | *T. yunnanensis* | Tezuka et al. (2011) |
|  | (7*S*,8*S*)-methoxy3′,7-epoxy-8,4′-oxyneoligna-4,9,9′-triol | Taxane | *T. yunnanensis* | Tezuka et al. (2011) |
|  | R-conidendrin | Taxane | *T. yunnanensis* | Tezuka et al. (2011) |
|  | Taxuyunnanine S | Taxane | *T. yunnanensis* | Li et al. (2002a) |
|  | Taxuyunnanine T | Taxane | *T. yunnanensis* | Li et al. (2002a) |
|  | Taxuyunnanine U | Taxane | *T. yunnanensis* | Li et al. (2002a) |
|  | Taxuyunnanine V | Taxane | *T. yunnanensis* | Li et al. (2002a) |
|  | Taxayunnin | Taxane | *T. yunnanensis* | Li et al. (2002a) |
|  | Taxamairin C | Taxane | *T. yunnanensis* | Li et al. (2002a) |
|  | Taxamairin A | Taxane | *T. yunnanensis* | Li et al. (2002a) |
|  | 3b-Hydroxysandaracopimaric Acids | Taxane | *T. yunnanensis* | Li et al. (2002a) |
|  | (1)-3-Hydroxyisodrimenin | Taxane | *T. yunnanensis* | Li et al. (2002a) |
|  | Dantaxusin C | Taxane | *T. yunnanensis* | Shinozaki et al. (2002) |
|  | Dantaxusin D | Taxane | *T. yunnanensis* | Shinozaki et al. (2002) |
|  | Taxuyunnanine G | Taxane | *T. yunnanensis* | Zhang et al. (1995) |
|  | Taxuyunnanine H | Taxane | *T. yunnanensis* | Zhang et al. (1995) |
|  | Taxuyunnanine I | Taxane | *T. yunnanensis* | Zhang et al. (1995) |
|  | Taxuyunnanine J | Taxane | *T. yunnanensis* | Zhang et al. (1995) |
|  | 1*β*-hydroxy-7-deacetyl-baccatin I | Taxane | *T. yunnanensis* | Zhang et al. (1997) |
|  | 1*β*-hydroxy-9-deacetyl-baccatin I | Taxane | *T. yunnanensis* | Zhang et al. (1997) |
|  | 1*β*-hydroxy-10-deacetyl-baccatin I | Taxane | *T. yunnanensis* | Zhang et al. (1997) |
|  | 10- deacetyl-baccatin VI | Taxane | *T. yunnanensis* | Zhang et al. (1997) |
|  | 9-deacetyl-baccatin VI | Taxane | *T. yunnanensis* | Zhang et al. (1997) |
|  | Taxayuntin H | Taxane | *T. yunnanensis* | Zhou et al. (1998) |
|  | Taxayuntin J | Taxane | *T. yunnanensis* | Zhou et al. (1998) |
|  | 10-acetoxy-2,5,7,9-tetrahydroxytaxa-4(20)，11- dien-13-one | Taxane | *T. yunnanensis* | Nguyen et al. (2003) |
|  | 2a-acetoxy-9a-benzoyloxy-5a,7b,10b,15- tetrahydroxy-11(15→1)-abeotaxa-4(20),11-dien-13-one | Taxane | *T. yunnanensis* | Nguyen et al. (2003) |
|  | 1b-acetoxy-7-drimen-11a-ol-12,11-lactone | Taxane | *T. yunnanensis* | Nguyen et al. (2003) |
|  | 1b-acetoxy-11,12-epoxy-6-drimen-8a,11a-diol | Taxane | *T. yunnanensis* | Nguyen et al. (2003) |
|  | Taxayuntin E | Taxane | *T. yunnanensis* | Yue et al. （1995) |
|  | Taxayuntin F | Taxane | *T. yunnanensis* | Yue et al. （1995) |
|  | 7b-Xylosyl-taxol D | Taxane | *T. yunnanensis* | Li et al. （2001) |
|  | Taxuyuntin G | Taxane | *T. yunnanensis* | Li et al. （2001) |
|  | Taxuyunnanine P | Taxane | *T. yunnanensis* | Li et al. （2001) |
|  | Taxuyunnanine Q | Taxane | *T. yunnanensis* | Li et al. （2001) |
|  | Taxuyunnanine R | Taxane | *T. yunnanensis* | Li et al. （2001) |
|  | Taxuyunnanine K | Taxane | *T. yunnanensis* | Li et al. （2000) |
|  | Taxuyunnanine L | Taxane | *T. yunnanensis* | Li et al. （2000) |
|  | Taxuyunnanine M | Taxane | *T. yunnanensis* | Li et al. （2000) |
|  | Taxuyunnanine N | Taxane | *T. yunnanensis* | Li et al. （2000) |
|  | Taxuyunnanine O | Taxane | *T. yunnanensis* | Li et al. （2000) |
|  | Dantaxusin A | Taxane | *T. yunnanensis* | Shinozaki et al. （2001) |
|  | Dantaxusin B | Taxane | *T. yunnanensis* | Shinozaki et al. （2001) |
|  | Baccatin VIII. | Taxane | *T. yunnanensis* | Hai et al. （2014) |
|  | Baccatin IX | Taxane | *T. yunnanensis* | Hai et al. （2014) |
|  | Baccatin X | Taxane | *T. yunnanensis* | Hai et al. （2014) |
|  | 7,9-dideacetyltaxayuntin | Taxane | *T. yunnanensis* | Zhong et al. （1996) |
|  | 9*α*,13*α*-diacetoxy-11(15→1)*abeotaxa*-4(20),11-diene-5*α*,10*β*,15-triol | Taxane | *T. yunnanensis* | Shi et al. （1999) |
|  | 9*α*,13*α*-diacetoxy-10*β*-benzoyloxy-5*α*-(3'-dimethyl-amino-3'-phenyl)-propionyloxy-11(15→1)abeotaxa-4(20),11-diene-15-ol | Taxane | *T. yunnanensis* | Shi et al. （1999) |
|  | 2*α*,7*β*,13*α*-triacetoxy-10*β*-hydroxy-5*α*-(3'-dimethylamino-3' -phenyl)-propionyl-oxy-2(3-20)-abeo-taxa-9-one | Taxane | *T. yunnanensis* | Shi et al. （1999) |
|  | 1*β*,10*β*-dihydroxy-9*α*,13*α*-diacetoxy-5*α*-(3'-dimethylamino-3’-phenyl)-propionyloxytaxa-4(20),11-diene | Taxane | *T. yunnanensis* | Shi et al. （1999) |
|  | 5*α*-hydroxy-9*α*,10*β*,13*α*-triacetoxy-11(15→1)abeotaxa-4(20),11-diene | Taxane | *T. yunnanensis* | Shi et al. （1999) |
|  | 5*α*,13*α*-dihydroxy-9*α*,10*β*-diacetoxy-11(15→1)abeotaxa-4(20),11-diene | Taxane | *T. yunnanensis* | Shi et al. （1999) |
|  | Sappanchalcone | Sappanols | *C. sappan* | Zhou (2017) |
|  | 3-deoxysappanchalcone | Sappanols | *C. sappan* | Wang and Liang (2016) |
|  | 2'-hydroxy-3，4，4'-trimethoxychalcone | Sappanols | *C. sappan* | Wang et al. (2003) |
|  | 4，4'-dihydroxy-2'-methoxychalcone | Sappanols | *C. sappan* | Wang et al. (2003) |
|  | 3，2'，4'-trihydroxyl-4-methoxychalcone | Sappanols | *C. sappan* | Cai (2012) |
|  | ( *αR*) -*α*，3，4，2'，4'-pentahydroxydihydrochalcone | Sappanols | *C. sappan* | Wang et al. (2010) |
|  | 2'，4'，3，4-tetrahydroxy-*β*-hydroxychalcone | Sappanols | *C. sappan* | Wang (2006) |
|  | Cephasinenoside A | Cephalotane diterpenoid glucoside | *C. sinensis* | Zhao et al. (2019) |
|  | Cephalotanins A | Norditerpenoids | *C. sinensis* | Xu et al. (2016) |
|  | Cephalotanins B | Norditerpenoids | *C. sinensis* | Xu et al. (2016) |
|  | Cephalotanins C | Norditerpenoids | *C. sinensis* | Xu et al. (2016) |
|  | Cephalotanins D | Norditerpenoids | *C. sinensis* | Xu et al. (2016) |
|  | Cephasinene A | Bisabolane sesquiterpenoid | *C. sinensis* | Ahmed et al. (2018) |
|  | Cephasinene B | Abietane diterpenoid | *C. sinensis* | Ahmed et al. (2018) |
|  | Angustanoic acid E | Abietanes | *C. sinensis* | Ahmed et al. (2018) |
|  | Epipalustric acid | Abietanes | *C. sinensis* | Ahmed et al. (2018) |
|  | Lambertianic acid | Labdane diterpenoid | *C. sinensis* | Ahmed et al. (2018) |
|  | Grasshopper ketone. | Megastigmane sesquiterpenoids | *C. sinensis* | Ahmed et al. (2018) |
|  | Vomifoliol | Megastigmane sesquiterpenoids | *C. sinensis* | Ahmed et al. (2018) |
|  | Allohydroxymatairesino | Lignans | *C. sinensis* | Ahmed et al. (2018) |
|  | Hydroxymatairesinol | Lignans | *C. sinensis* | Ahmed et al. (2018) |
|  | (+)-nortrachelogenin | Lignans | *C. sinensis* | Ahmed et al. (2018) |
|  | Tanegool | Lignans | *C. sinensis* | Ahmed et al. (2018) |
|  | (7*S*,8*R*)-dihydro-3′-hydroxy-8-hydroxy-methyl-  7-(4-hydroxy-3-methoxyphenyl)-1′- benzofuranpropanol | Lignans | *C. sinensis* | Ahmed et al. (2018) |
|  | Threo-guaiacylglycerol-*β*-*O*-4′-dihydroconiferyl ether | Lignans | *C. sinensis* | Ahmed et al. (2018) |
|  | Spiramongolin | Spiramongolin | *C. sinensis* | Ahmed et al. (2018) |
|  | Pseudolarifuroic acid | Lannostane-type tritepene | *C. sinensis* | Ahmed et al. (2018) |
|  | Caprylic acid methyl ester | Ester | *A. catechu* | Thakur et al. (2018) |
|  | 2-Ethyl-3-methyl-  1-butene | Ester | *A. catechu* | Thakur et al. (2018) |
|  | 4-Hydroxybenzoic  acid | Acid | *A. catechu* | (Adhikari et al., 2021) |
|  | Myristic acid methyl ester | Ester | *A. catechu* | Thakur et al. (2018) |
|  | Lauric acid methyl ester | Ester | *A. catechu* | Thakur et al. (2018) |
|  | 4-Hydroxyphenyl ethanol | Phenolic | *A. catechu* | Li et al. （2011) |
|  | Gallic acid | Phenolic acids | *A. catechu* | Adhikari et al. (2021) |
|  | Chlorogenic acid | Acid | *A. catechu* | Adhikari et al. (2021) |
|  | Umbelliferone | Other compounds | *A. catechu* | Adhikari et al. (2021) |
|  | Coumaric acid | Acid | *A. catechu* | Adhikari et al. (2021) |
|  | Caffeic acid | Acid | *A. catechu* | Adhikari et al. (2021) |
|  | Camphor | Other compounds | *A. catechu* | Adhikari et al. (2021) |
|  | Phytol | Alkanol | *A. catechu* | Adhikari et al. (2021) |
|  | Hexadecane | Other compounds | *A. catechu* | Adhikari et al. (2021) |
|  | Vitamin E acetate | Other compounds | *A. catechu* | Adhikari et al. (2021) |
|  | Rubrosterone | Steroids | *T. yunnanensis* | Li et al. (2002b) |
|  | Ponasterone A | Steroids | *T. yunnanensis* | Li et al. (2002b) |
|  | Ecdysterone | Steroids | *T. yunnanensis* | Li et al. (2002b) |
|  | 20-Hydroxyechysone- 20,22-monoacetonide | Steroids | *T. yunnanensis* | Li et al. (2002b) |
|  | 7-Oxositosterol | Steroids | *T. yunnanensis* | Li et al. (2002b) |
|  | Stigmast-4-en-6*β*-ol-3-one | Steroids | *T. yunnanensis* | Li et al. (2002b) |
|  | 5*α*,6*β*-Dihydroxy-daucosterol | Steroids | *T. yunnanensis* | Li et al. (2002b) |
|  | Taxuyunins A | Lignans | *T. yunnanensis* | Li et al. (2003b) |
|  | Taxuyunins B | Lignans | *T. yunnanensis* | Li et al. (2003b) |
|  | Brevitaxin | Lignans | *T. yunnanensis* | Li et al. (2003b) |
|  | Caesalsappanin R | Cassane Diterpenoids | *C. sappan* | Zhu et al. (2017) |
|  | Caesalsappanin S | Cassane Diterpenoids | *C. sappan* | Zhu et al. (2017) |
|  | Caesalsappanins A | Cassane Diterpenoids | *C. sappan* | Ma et al. (2015) |
|  | Caesalsappanins B | Cassane Diterpenoids | *C. sappan* | Ma et al. (2015) |
|  | Caesalsappanins C | Cassane Diterpenoids | *C. sappan* | Ma et al. (2015) |
|  | Caesalsappanins D | Cassane Diterpenoids | *C. sappan* | Ma et al. (2015) |
|  | Caesalsappanins E | Cassane Diterpenoids | *C. sappan* | Ma et al. (2015) |
|  | Caesalsappanins F | Cassane Diterpenoids | *C. sappan* | Ma et al. (2015) |
|  | Caesalsappanins G | Cassane Diterpenoids | *C. sappan* | Ma et al. (2015) |
|  | Caesalsappanins H | Cassane Diterpenoids | *C. sappan* | Ma et al. (2015) |
|  | Caesalsappanins I | Cassane Diterpenoids | *C. sappan* | Ma et al. (2015) |
|  | Caesalsappanins J | Cassane Diterpenoids | *C. sappan* | Ma et al. (2015) |
|  | Caesalsappanins K | Cassane Diterpenoids | *C. sappan* | Ma et al. (2015) |
|  | Caesalsappanins L | Cassane Diterpenoids | *C. sappan* | Ma et al. (2015) |
|  | Drupacine | Alkaloids | *C. sinensis* | Ma et al. (2016) |
|  | 11-Hydroxycephalotaxine | Alkaloids | *C. sinensis* | Ma et al. (2016) |
|  | Cephalancetine A | Alkaloids | *C. sinensis* | Ma et al. (2016) |
|  | Isocephalotaxine | Alkaloids | *C. sinensis* | Ma et al. (2016) |
|  | Cephalotaxine β-N-oxide | Alkaloids | *C. sinensis* | Ma et al. (2016) |
|  | 4-Hydroxycephalotaxine | Alkaloids | *C. sinensis* | Ma et al. (2016) |
|  | Wilsonine | Alkaloids | *C. sinensis* | Ma et al. (2016) |
|  | Cephalotaxine | Alkaloids | *C. sinensis* | Ma et al. (2016) |
|  | Phanginin R | Cassane Diterpenoids | *C. sappan* | Bao et al. (2016) |
|  | Protosappanin C | Lignans | *C. sappan* | Mueller et al. (2016) |
|  | Protosappanin B | Lignans | *C. sappan* | Mueller et al. (2016) |
|  | Sappanol | Lignans | *C. sappan* | Mueller et al. (2016) |
|  | α-conidendrin | Polyphenolics | *T. yunnanensis* | Hafezi et al. (2020) |
|  | Phanginin I | Cassane Diterpenoids | *C. sappan* | Tran et al. （2015) |
|  | Phaginin A | Cassane Diterpenoids | *C. sappan* | Tran et al. （2015) |
|  | Phanginin D | Cassane Diterpenoids | *C. sappan* | Tran et al. （2015) |
|  | Phanginin H | Cassane Diterpenoids | *C. sappan* | Tran et al. （2015) |
|  | Phanginin J | Cassane Diterpenoids | *C. sappan* | Tran et al. （2015) |

## **Table 6** Barrigenol triterpenoids from *Xanthoceras sorbifolium* Bunge.

| No | Compound | R1 | R2 | R3 | R4 | R5 | R6 | R7 | R8 | Ref. |
| --- | --- | --- | --- | --- | --- | --- | --- | --- | --- | --- |
|  | Mother nucleus A |  |  |  |  |  |  |  |  |  |
| 121 | 21,22-di-*O*-angeloyl-R1-barrigenol | H | CH_3_ | CH_3_ | OH | OH | H | Ang | Ang | Chen et al. (1984) |
| 122 | 21,22-di-*O*-angeloyl-24-hydroxy-R1-barrigenol | H | CH_2_OH | CH_3_ | OH | OH | H | Ang | Ang | Li (2006) |
| 123 | 21-*O*-angeloyl-R1-barrigenol | H | CH_3_ | CH_3_ | OH | OH | H | Ang | H | Li (2004) |
| 124 | 22-*O*-angeloyl-R1-barrigenol | H | CH_3_ | CH_3_ | OH | OH | H | H | Ang | Buren (2007) |
| 125 | R1-barrigenol | OH | CH_3_ | CH_3_ | OH | OH | H | H | H | Li (2006) |
| 126 | 3-*O*-(3-*O*-*α*-L-arabinofuranosyl-2-*O*-*β*-D-galactopyranosyl)- *β*-*O*-glucopyranosyl-21, 22-di-*O*-angeloyl-R1-barrigenol | R’ | CH_3_ | CH_3_ | OH | OH | CH_2_OH | Ang | Ang | Li (2006) |
| 127 | 22-*O*-angeloyl-21-*O*-epoxyangeloyl-barringtogenol C | H | CH_3_ | CH_3_ | H | OH | H | epoxyangeloyl | Ang | Li (2006) |
| 128 | 21, 22-di-*O*-angeloyl-barringtogenol C | H | CH_3_ | CH_3_ | H | OH | H | Ang | Ang | Li (2006) |
| 129 | Barringtogenol C | H | CH_3_ | CH_3_ | H | OH | H | H | H | Li (2006) |
| 130 | 16-deoxybarringtogenol C | H | CH_3_ | CH_3_ | H | H | H | H | H | Li (2006) |
| 131 | 28-*O*-*β*-D-glucopyranosyl-16-deoxybarringtogenol | H | CH_3_ | CH_3_ | H | H | Glc | H | H | Li (2006) |
| 132 | 16-*O*-acetyl-21-*O*-(4-*O*-angeloyl-*α*-L-rhamnopytanosyl)- barringtogenol C | H | CH_3_ | CH_3_ | H | OAc | H | A | H | Li (2006) |
| 133 | 3-*O*-*β*-D-glucopyranosyl(1→6)-*β*-D-glucopyranosyl-28-*O*-*β*-D-glucopyranosyl(1→6)[*α*-L-rhamnopytanosyl (1→ 2)]-*β*-D-glucopyranosyl-16-deoxybarringtogenol C | Glc (1→6) Glc | CH_3_ | CH_3_ | H | H | B | H | H | Li et al.(2006) |
| 134 | 3-*O*-[*β*-D-glucopyranosyl-(1→6)](3′-*O*-angeloyl)-*β*-D-glucopyranosyl-28-*O*-[*α*-L-rhamnopytanosyl-(1→2)]-*β*-D-glucopyranosyl-16- deoxybarringtogenol C | C | CH_3_ | CH_3_ | H | H | Glc (2→1) Rha | H | H | Li et al.(2008) |
| 135 | 3-*O*-*β*-D-glucopyranosyl-16-deoxybarringtogenol C | Glc | CH_3_ | CH_3_ | H | H | H | H | H | Wang et al. (2011) |
| 136 | 16-*O*-acetyl-21-*O*-(3′,4′-di-*O*-angeloyl)-*β*-D-fucopyranosyl theasapogenol B | H | CH_3_ | CH_3_ | H | OAc | H | D | H | (Li et al., 2007c) |
| 137 | 22-*O*-acetyl-21-*O*-(4′-*O*-angeloyl)-*β*-D-fucopyranosyl theasapogenol B | H | CH_3_ | CH_3_ | H | OH | H | D | Ac | Li et al.(2006) |
| 138 | 28-*O*-*β*-D-glucopyranosyl-21-*O*-angeloyl-R1-barrigenol | H | CH_3_ |  |  | OH | CH_3_ | OH | H | Wang et al. (2011) |
| 139 | 21-*O*-angeloyl-24-hydroxy-R1-barrigenol | H | CH_2_OH | CH_3_ | OH | OH | H | Ang | H | (Li, 2008) |
| 140 | 3-*O*-(3-*O*-*α*-L-arabinofuranosyl-2-*O*-*β*-D-galactopyranosyl)-(6-*O*-methyl)-*β*-D-glucuronopyranosyl-21, 22-di-*O*-angeloyl-R1-barrigenol | R’ | CH_3_ | CH_3_ | OH | OH | H | Ang | Ang | Wang et al. (2011) |
| 141 | 3-*O*-[*β*-D-galactopyranosyl(1→2)-*α*-L-arabinofuranosyl (1→3)-*β*-D-methylglucuronic-acid-21-*O*-(3,4-diangeloyl)-*α*-L-rhamnose-3*β*, 16*α*,21*β*, 22*α*, 28*β*-pentahydroxyl-22-acetoxy-olean12-ene | E | CH_3_ | CH_3_ | H | OH | H | F | Ac | Guo et al.(2009) |
| 142 | 3-*O*-*β*-D-galactopyranosyl-(1→6)-(2-*O*-angeloyl)-*β*-D-glucopyranosyl saniculagenic C-28-*O*-*α*-L-rhamnopytanosyl-(1→2)-*β*-D-glucopyranoside | G | CH_3_ | CH_3_ | OH | OH | Rha (1→2) Glc | H | H | Fu et al. (2010) |
| 143 | Xanifolia Y_0_ | R_1_ | H | CH_3_ | OH | OH | H | Ang | i-Bu | Chan et al. (2008) |
| 144 | Xanifolia Y_2_ | M | OH | CH_3_ | OH | OH | H | Ang | Ang | Chan et al. (2008) |
| 145 | Xanifolia Y_3_ | R’ | H | CH_3_ | OH | OH | H | Ang | Ang | Chan et al. (2008) |
| 146 | Xanifolia Y_7_ | R’ | H | CH_3_ | OH | OH | 2-methylbutanoyl | Ang | H | Chan et al. (2008) |
| 147 | Sorbifoliumside G | Glc (1→6) Glc | CH_3_ | CH_3_ | H | =0 | I | H | H | Yu et al. (2012b) |
| 148 | Sorbifoliumside H | J | CH_3_ | CH_3_ | H | =0 | I | H | H | Yu et al. (2012b) |
| 149 | Sorbifoliumside I | Glc (1→6) Glc | CH_3_ | CH_3_ | H | H | Glc (1→6) Glc | H | H | Yu et al. (2012b) |
| 150 | Sorbifoliumside J | J | CH_3_ | CH_3_ | H | =0 | Glc (1→6) Glc | H | H | Yu et al. (2012b) |
| 151 | 3-*O*-*β*-D-glucopyranosyl-28-*O*-[*β*-D-glucopyranosyl (1→2)]-*β*-D-glucopyranosyl-21*β*, 22*α*- dihydroxyl-olean-12-ene | Glc | H | H | H | H | Glc (1→2) Glc | H | H | Cui et al. (2012) |
| 152 | Sorbifoliumsides A | Glc (1→6) Glc | H | H | H | H | R’’ | H | H | Yu et al. (2012b) |
| 153 | Sorbifoliumsides B | Glc (1→6) Glc | H | H | H | =0 | R’’ | H | H | Yu et al. (2012b) |
| 154 | Sorbifoliumsides C | R’’ | H | H | H | =0 | Glc (1→6) Glc | H | H | Yu et al. (2012b) |
| 155 | Sorbifoliumsides D | R’’’ | H | H | H | H | Glc (1→6) Glc | H | H | Yu et al. (2012b) |
| 156 | Sorbifoliumsides E | R’’’ | H | H | H | =0 | Glc (1→6) Glc | H | H | Yu et al. (2012b) |
| 157 | Sorbifoliumsides F | J | H | H | H | H | R’’ | H | H | Yu et al. (2012b) |
| 158 | Bunkankasaponin F | K | H | H | H | OH | H | L | Ac | Yu et al. (2012b) |
| 159 | 3-*O*-*β*-D-glucopyranosyl (1→6)-[angeloyl (1→ 2)]-*β*-D-glucopyranosyl-28-*O*-*α*-L-rhamnopyranosyl (1→2)-[*β*-D-glucopyranosyl (1→6)]- *β*-D-glucopyranosyl-21*β*, 22*α*-dihydroxylolean-12-ene | C | H | H | H | H | M | H | H | Cui et al. (2012) |
| 160 | 3-*O*-*β*-D-glucopyranosyl,28-*O*-[*α*-L-rhamnosyl (1→2)]-*β*-D- glucopyranosyl-16- deoxybarringtogenol C | Glc | CH_3_ | CH_3_ | H | H | Glc (1→6) Glc | H | H | Li et al.(2008) |
| 161 | 3-*O*-[*β*-D-galactopyranosyl (1→2)]-*α*-L-arabinofuranosyl (1→3)-*β*-D-methyl glucuronic acid21, 22-*O*-diangeloyl-3*β*, 15*α*, 16*α*, 21*β*, 22*α*, 28*β*-hexahydroxyl-olean-12-ene | E | CH_3_ | CH_3_ | OH | OH | H | Ang | Ang | Guo et al.(2009) |
| 162 | Mother nucleus B  22-*O*-acnapoleogenin B | GlcUA | OH | H | H | Ac | Ang | Ang |  | Chen et al. （1985c) |
| 163 | Napoleogenin B | H | OH | H | H | H | Ang | Ang |  | Chen et al. （1985c) |
| 164 | 21-*O*-(3, 4-di-*O*-angeloyl)-*β*-D-fucopyranosyl theasapogenol B | H | H | H | H | H | Ang | Ang |  | Chen et al. （1985c) |
| 165 | 21-*O*-(4-*O*-acetyl-3-*O*-angeloyl)-*β*-D-fucopyranosyl theasapogenol B | H | H | H | H | H | Ang | Ac |  | Chen et al. （1985c) |
| 166 | 21-*O*-(4-*O*-acetyl-3-*O*-angeloyl)-*β*-D-fucopyranosyl-22-*O*-acetyl protoaescigenin | H | OH | H | H | Ac | Ang | Ac |  | Chen et al. （1985c) |
| 167 | 16-*O*-acetyl-21-*O*-(3, 4-di-*O*-angeloyl)-*β*-Dfucopyranosyl protoaescigen | H | OH | Ac | H | H | Ang | Ang |  | Chen et al. （1985c) |
| 168 | 3-*O*-*β*-D-glucuronopyranoside bunkankasaponin A | Glc (1→2) GlcUA | OH | H | H | Ac | Ang | Ac |  | Chen et al. （1985c) |
| 169 | Bunkankasaponin B | Glc (1→2) GlcUA | OH | H | H | Ac | Ang | Ang |  | Chen et al. （1985c) |
| 170 | Bunkankasaponin C | Glc (1→2) GlcUA | OH | H | Ac | H | Ang | Ac |  | Chen et al. （1985c) |
| 171 | Bunkankasaponin D | Glc (1→2) GlcUA | OH | H | Ac | H | Ang | Ang |  | Chen et al. （1985c) |

## **Table 7** Lupane triterpenoids (C) from *Xanthoceras sorbifolium* Bunge.

| No | Compound | R_1_ | R_2_ | R_3_ | R_4_ | Reference |
| --- | --- | --- | --- | --- | --- | --- |
| 172 | 3*β*,23-dihydroxy-lup-20(29)-en-28-oic acid23-caffeate | H | N | H | COOH | Li et al. (2007b) |
| 173 | 3*β*,23-dihydroxy-lup-20(29)-en-28-oic acid3-caffeate | N | OH | H | COOH | Li (2008) |
| 174 | Betulin | H | H | H | CH_2_OH | Li (2008) |
| 175 | 23-hydroxybetulinic acid | H | H | OH | COOH | Li (2008) |

## **Table 8** Tirucallane triterpenoids (D) from *Xanthoceras sorbifolium* Bunge.

| NO | Compound | R_1_ | R_2_ | R_3_ | Reference |
| --- | --- | --- | --- | --- | --- |
| 176 | 3*β*-hydroxytirucalla-7, 24-dien-21-oic acid | OH | H | CH_3_ | Ma et al. (2000) |
| 177 | 3*α*, 29-dihydroxytirucalla-7, 24-dien-21-oic acid | H | OH | CH_2_OH | Ma et al. (2000) |
| 178 | 3*β*, 29-dihydroxytirucalla-7, 24-dien-21-oic acid | OH | H | CH_2_OH | Ma et al. (2000) |
| 179 | 3-oxotriucalla-7, 24-dien-21-oic acid | ＝O | _ | CH_3_ | Ma et al. (2000) |
| 180 | 29-hydroxy-3-oxotirucalla-7, 24-dien-21-oic acid | ＝O | _ | CH_2_OH | Ma et al. (2000) |
| 181 | 29-O-acetyl-3-oxotirucalla-7, 24-dien-21-oic acid | ＝O | _ | CH_2_OCOCH_3_ | Ma et al. (2000) |
